# Integrator-Dependent and Allosteric/Intrinsic Mechanisms Ensure Efficient Termination of snRNA Transcription

**DOI:** 10.1016/j.celrep.2020.108319

**Published:** 2020-10-27

**Authors:** Lee Davidson, Laura Francis, Joshua D. Eaton, Steven West

**Affiliations:** 1 The Living Systems Institute, University of Exeter, Stocker Rd, Exeter EX4 4QD, UK

**Keywords:** snRNA, transcription termination, Integrator, exosome, RNA polymerase, cleavage, polyadenylation, INTS11, XRN2, allosteric

## Abstract

Many RNA polymerases terminate transcription using allosteric/intrinsic mechanisms, whereby protein alterations or nucleotide sequences promote their release from DNA. RNA polymerase II (Pol II) is somewhat different based on its behavior at protein-coding genes where termination additionally requires endoribonucleolytic cleavage and subsequent 5′→3′ exoribonuclease activity. The Pol-II-transcribed small nuclear RNAs (snRNAs) also undergo endoribonucleolytic cleavage by the Integrator complex, which promotes their transcriptional termination. Here, we confirm the involvement of Integrator but show that Integrator-independent processes can terminate snRNA transcription both in its absence and naturally. This is often associated with exosome degradation of snRNA precursors that long-read sequencing analysis reveals as frequently terminating at T-runs located downstream of some snRNAs. This finding suggests a unifying vulnerability of RNA polymerases to such sequences given their well-known roles in terminating Pol III and bacterial RNA polymerase.

## Highlights

• Integrator-dependent and -independent mechanisms terminate snRNA transcription
• Integrator-independent termination is allosteric/intrinsic in nature
• Sites of allosteric/intrinsic termination often coincide with T-runs
• Some Pol II termination may share features with Pol III/prokaryotic mechanisms

## Graphical Abstract

**Figure:**
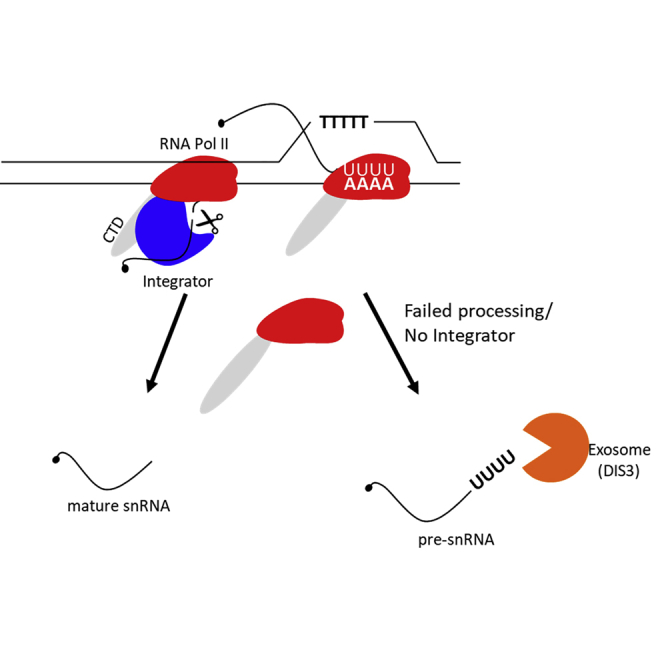

## Teaser

Davidson et al. show that termination of RNA polymerase II transcription at human snRNA genes can occur by Integrator-dependent and -independent pathways. The latter uses an allosteric/intrinsic mechanism most efficiently detected where T-runs are present in the non-template strand. This is reminiscent of the way many other RNA polymerases terminate.

## Introduction

Termination of RNA polymerase II (Pol II) on protein-coding genes requires a polyadenylation signal (PAS) that is bound and endoribonucleolytically processed by a multi-protein cleavage and polyadenylation (CPA) complex with catalytic activity supplied by CPSF73 (Mandel et al., 2006; Proudfoot, 2011, 2016). CPSF73 is crucial because its depletion induces transcriptional read-through that is often hundreds of kilobases in length (Eaton et al., 2020). Its Pol-II-associated cleavage product is degraded 5′→3′ by XRN2, which promotes termination by the so-called “torpedo” model (Eaton et al., 2018; Fong et al., 2015). This mechanism also incorporates allosteric features, defined as modifications to the elongation complex, which slow polymerases down at the end of the gene (Cortazar et al., 2019; Eaton et al., 2020; Eaton and West, 2020). However, Pol II transcribes multiple gene classes where transcriptional termination mechanisms are relatively unexplored. In many of these cases, primary transcripts are endoribonucleolytically cleaved, suggesting some common mechanistic features (Baillat et al., 2005; Fatica et al., 2000).

The spliceosomal small nuclear RNAs (snRNAs) are a prominent Pol-II-transcribed gene class. snRNA precursors are endoribonucleolytically cleaved by the Integrator complex, in which a paralog of CPSF73, called INTS11, provides endoribonuclease activity and promotes transcriptional termination (Baillat et al., 2005; O’Reilly et al., 2014; Skaar et al., 2015). Cleavage is positioned by a 3′ box sequence located after the cut site (Guiro and Murphy, 2017). Recent findings implicate Integrator in promoter-proximal regulation of Pol II transcription (Beckedorff et al., 2020; Elrod et al., 2019; Stadelmayer et al., 2014; Tatomer et al., 2019). Integrator is also involved in the termination of other non-coding transcripts, including enhancer RNAs and promoter upstream transcripts (Beckedorff et al., 2020; Lai et al., 2015; Nojima et al., 2018). Lastly, Integrator mutations are associated with some neurological diseases (Oegema et al., 2017).

In budding yeast, termination of protein-coding gene transcription is similar to that in humans (Kim et al., 2004; West et al., 2004). However, snRNA termination uses the Nrd1-Nab3-Sen1 (NNS) complex, which releases precursors that are then subject to 3′→5′ degradation (Allmang et al., 1999; Schaughency et al., 2014; Steinmetz et al., 2001). Termination of many other RNA polymerases is not associated with prior endo- or exoribonuclease activity and defines 3′ ends directly. For example, Pol III terminates at four or more Ts in the non-template strand, and the prokaryotic RNA polymerase often terminates at similar sequence elements aided by an upstream hairpin sequence (Arimbasseri et al., 2014; Ray-Soni et al., 2016; Zenkin, 2014). The weak thermodynamic stability of rU:dA hybrids may help this process (Martin and Tinoco, 1980). Interestingly, T-runs were identified decades ago as intrinsic terminators of purified human Pol II *in vitro* (Dedrick et al., 1987; Reines et al., 1987).

We have investigated the transcriptional termination mechanism on snRNA genes by asking whether there is obligate coupling to Integrator activities. There is clear involvement of Integrator, but termination still occurs without it. In some cases, this involves CPSF73; however, an alternative process seems to directly release 3′ ends for exosome degradation. The characterization of exosome-targeted snRNAs reveals stochastic termination without upstream cleavage and sometimes at T-runs. This feature suggests a common underlying principle for transcriptional termination that, for Pol II, can be applied at snRNA genes.

## Results and Discussion

### INTS11 Depletion Delays but Does Not Abolish Termination of snRNA Transcription

**Figure 1:**
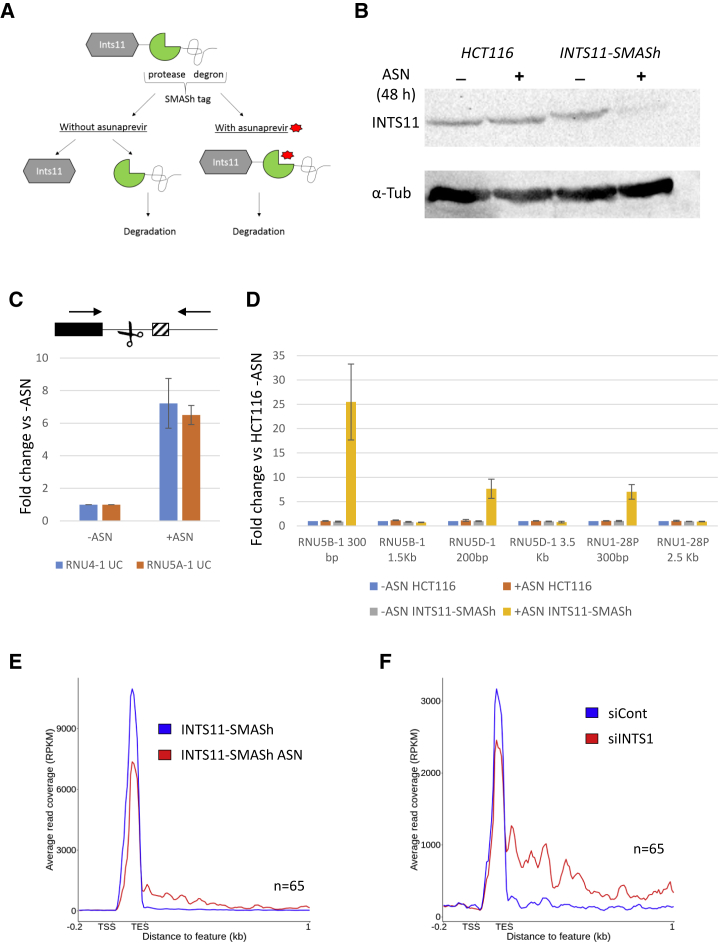
Termination of snRNA Transcription Is Partially Impaired by Integrator Depletion (A) Schematic of the SMASh tag system. Under normal conditions, the degron-encoded NS3 protease (green) removes the tag and the protein is stable. Addition of ASN (red) prevents this promoting proteosomal degradation. (B) Western blot demonstrating ASN-dependent depletion of INTS11 after 48 h. Unmodified cells are shown as a control and the lower tubulin blot shows loading. (C) qRT-PCR analysis of unprocessed (UC, uncleaved) RNU4-1 and RNU5A-1 transcripts in *INTS11-SMASh* cells treated or not with ASN. Levels in treated cells are shown relative to untreated cells after normalizing to spliced actin levels. The schematic shows snRNA (black rectangle), canonical Integrator cleavage site (scissors), downstream 3′ box (hatched box), and primers used (arrows). n = 4, error bars are standard error of the mean (SEM). (D) qRT-PCR of RNU5 and RNU1 variants in HCT116 or *INTS11-SMASh* cells treated or not with ASN. Primers were used to detect read-through downstream of the annotated 3′ ends of each gene, with the distance indicated under each set of bars. Values are expressed as a fold change versus untreated HCT116 cells after normalizing to spliced actin levels. n = 3, error bars are SEM. (E) Metagene plot of snRNA genes from nuclear RNA-seq performed on *INTS11-SMASh* cells treated or not with ASN. Signal is reads per kilobase of transcript, per million mapped reads (RPKM). TSS, transcription start site; TES, annotated snRNA 3′ end. (F) Metagene plot of read-through at all expressed snRNA genes in chromatin-associated RNA-seq from HCT116 cells treated with control or INTS1-specific siRNAs. Signal is RPKM.

To study the role of Integrator in snRNA transcriptional termination, we used CRISPR-Cas9 to Carboxyl (C)-terminally tag endogenous *INTS11* with a small-molecule-assisted shut-off (SMASh) modality (Chung et al., 2015). An internal NS3 protease constitutively cleaves off the SMASh tag but is inhibited by Asunaprevir (ASN) whereupon the tagged factor is degraded (Figure 1A). A constitutively removed tag was selected because the C-terminus of INTS11 is important for functional interactions (Albrecht and Wagner, 2012). HCT116 cells were edited due to their diploid karyotype. PCR confirmed homozygous targeting of *INTS11* with SMASh (Figures S1A and S1B), and western blotting demonstrated depletion of the INTS11 protein after 48 h of ASN treatment (Figure 1B).

We analyzed the impact of INTS11 depletion on snRNA processing by using quantitative reverse transcription and PCR (qRT-PCR) with primers spanning the canonical Integrator cleavage sites on RNU4-1 and RNU5A-1. These species accumulate following ASN treatment, confirming the effectiveness of the system (Figure 1C). Next, we analyzed extended transcripts at three other snRNA genes by using snRNA-proximal and -distal primers, with samples from unmodified HCT116 cells included as a control. ASN treatment increases the levels of snRNA-proximal amplicons in *INTS11-SMASh* cells but not in unmodified cells, showing the specificity of the system (Figure 1D). The effect is lower at downstream regions, suggesting that termination occurs in delayed fashion when INTS11 is depleted. Nuclear RNA sequencing (RNA-seq), performed in *INTS11-SMASh* cells, showed this to be generally true for other expressed snRNAs (Figure 1E). An analysis of published Pol II occupancy data confirmed that INTS11 depletion delays but does not abolish snRNA termination (Figure S1C; Stadelmayer et al., 2014).

### INTS1 Depletion Delays but Does Not Abolish snRNA Transcriptional Termination

Other Integrator components might be more critical for transcriptional termination than INTS11. To test this, we depleted INTS1 by RNAi—chosen as the largest subunit and separate from the cleavage module (Albrecht et al., 2018). We confirmed INTS1 protein depletion (Figure S1D) and sequenced chromatin-associated RNA from cells treated with control or INTS1 small interfering RNA (siRNAs). Meta-analysis shows that INTS1 depletion increases snRNA read-through but that the effect declines within 1 kb (Figure 1F). An analysis of individual snRNAs/small nucleolar RNAs (snoRNAs) confirms that read-through RNA levels diminish to control levels within a few kb (Figure S1E). This was also the case when *RNU4-2* read-through was assayed following INTS1 and INTS11 co-depletion (Figure S1F). Therefore, although efficient termination of snRNA transcription requires Integrator, other mechanisms can compensate for its depletion.

### The Exosome Degrades Precursor snRNAs

**Figure 2:**
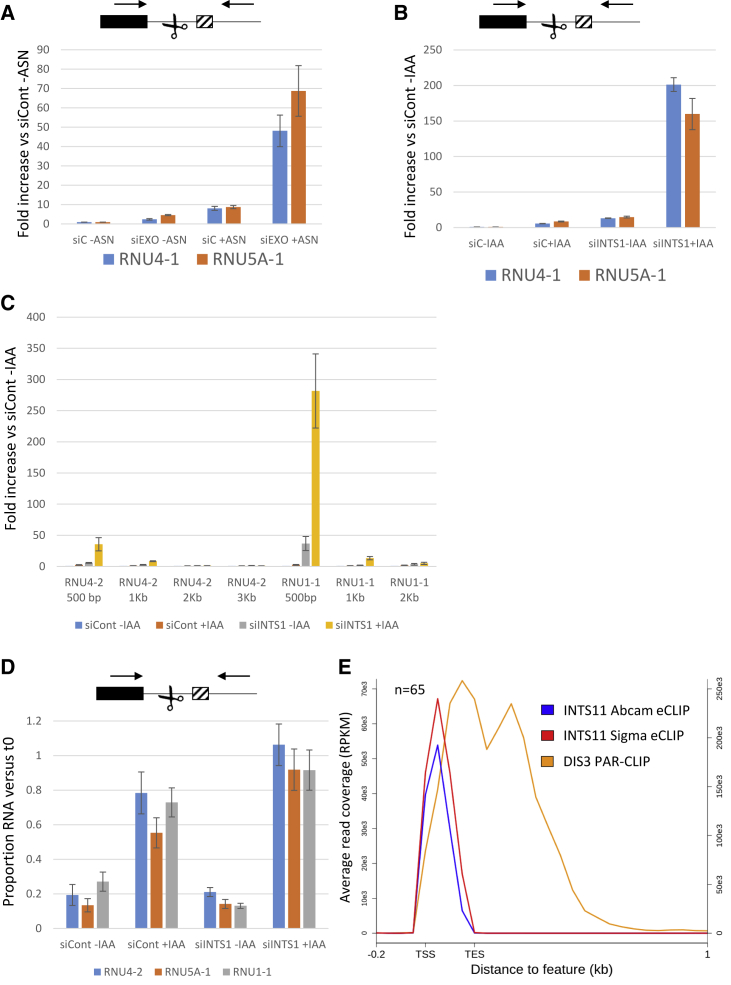
snRNA Precursors Are Targeted for 3′→5′ Degradation by the Exosome (A) qRT-PCR analysis of *INTS11-SMASh* cells transfected with control or EXOSC3-siRNAs before treatment or not with ASN. Unprocessed RNU4-1 and RNU5A-1 are quantitated relative to control siRNA transfected cells untreated with ASN (siC −ASN) after normalizing to spliced actin. n = 3, error bars are SEM. (B) qRT-PCR analysis of *DIS3-AID* cells transfected with control or INTS1 siRNAs before treatment or not with auxin (2 h). Unprocessed RNU4-1 and RNU5A-1 are shown relative to control siRNA transfected cells not treated with auxin (siC −IAA) after normalizing to spliced actin. n = 4, error bars are SEM. (C) qRT-PCR analysis of *DIS3-AID* cells transfected with control or INTS1 siRNAs before treatment or not with auxin (2 h). RNU1-1 and RNU4-2 extended read-through is quantitated relative to siCont-IAA samples after normalizing to spliced actin levels. n = 3, error bars are SEM. (D) qRT-PCR analysis of snRNA precursor stability in *DIS3-AID* cells transfected with control or INTS1 siRNAs before treatment or not with auxin. Quantitation shows the proportion of precursor remaining after 15 min of actD treatment versus the respective untreated condition (t0). n = 4, error bars are SEM. (E) Meta-analysis of DIS3 occupancy on snRNA genes as determined by PAR-CLIP performed in HEK293 cells (Szczepińska et al., 2015) and of INTS11 occupancy on snRNAs derived from published eCLIP in HeLa cells (Barra et al., 2020). Data from two different INTS11 antibodies are displayed. The y axes shows RPKM (left for DIS3, right for INTS11).

Integrator-independent termination should release 3′ ends that might be susceptible to degradation by the 3′→5′ exoribonucleolytic exosome. To investigate this, *INTS11-SMASh* cells were transfected with control siRNAs or siRNAs against the EXOSC3 subunit of the exosome before treatment or not with ASN to degrade INTS11. Unprocessed RNU4-1 and RNU5A-1 precursors accumulate following INTS11 loss as expected (Figure 2A). They also accumulate following EXOSC3 depletion, suggesting that some snRNA precursors are degraded by the exosome. Interestingly, co-depletion of EXOSC3 and INTS11 caused a much larger effect than their individual depletion. A similar result was seen following RNAi depletion of INTS1 and elimination of the catalytic subunit of the exosome DIS3 by an auxin-inducible degron (AID) tag (Figure 2B). Despite this large accumulation of precursor snRNAs following exosome and Integrator co-depletion, read-through distance remains limited, indicating continued transcriptional termination (Figure 2C). These data are supportive of exosome degradation of snRNA precursors that is more prominent upon reduced Integrator activity.

Another potential explanation for continued termination following Integrator depletion is that residual activity, remaining after knockdown, is sufficient to maintain an Integrator-dependent process. To test the functional extent of INTS1 depletion, *DIS3-AID* cells were transfected with control or INTS1 siRNAs before treatment or not with ASN. Transcription was then inhibited by actinomycin D (actD) for 15 min and unprocessed *RNU4-2*, *RNU5A-1*, and *RNU1-1* transcripts were monitored by qRT-PCR (Figure 2D). In control cells, actD caused a reduction in these precursors, reflecting continued processing/degradation following transcription inhibition. This reduction is less marked following DIS3 loss, which we confirmed using another method of transcriptional inhibition (Figure S2A). As primers span the Integrator cleavage sites, this suggests that DIS3 targets transcripts that escape maturation rather than competing with processing. This idea is supported by recent findings that mature snRNA accumulation is unaffected by exosome depletion (Lardelli and Lykke-Andersen, 2020). Following INTS1 depletion, a substantial fraction of precursors are lost after actD treatment either because of residual Integrator activity or degradation of the resulting unprocessed products. The latter explanation is favored because INTS1 and DIS3 co-depletion prevents most turnover following actD treatment (almost 100% of each precursor remains after 15 min of treatment). This level of functional loss makes it unlikely that residual Integrator fully accounts for termination following its depletion and implicates additional mechanisms.

The generation of precursor snRNA exosome substrates is likely to be common because 3′ flanking transcripts accumulate within just 1 h of DIS3 depletion (Figure S2B). Consistently, an analysis of published PAR-CLIP (photoactivatable ribonucleoside-enhanced crosslinking and immunoprecipitation) shows DIS3 occupancy of snRNAs 3′ flanking RNAs in otherwise unmodified cells (Figure 2E). In contrast, published eCLIP (enhanced CLIP) shows no clear INTS11 crosslinking beyond annotated snRNAs, suggesting that Integrator-independent processes may generate DIS3 substrates (Figure 2E). DIS3 does not occupy transcripts downstream of protein-coding genes, which are unaffected by its loss (Figure S2C; Davidson et al., 2019).

### Many Exosome-Targeted snRNA Precursors Are Released by Transcriptional Termination

**Figure 3:**
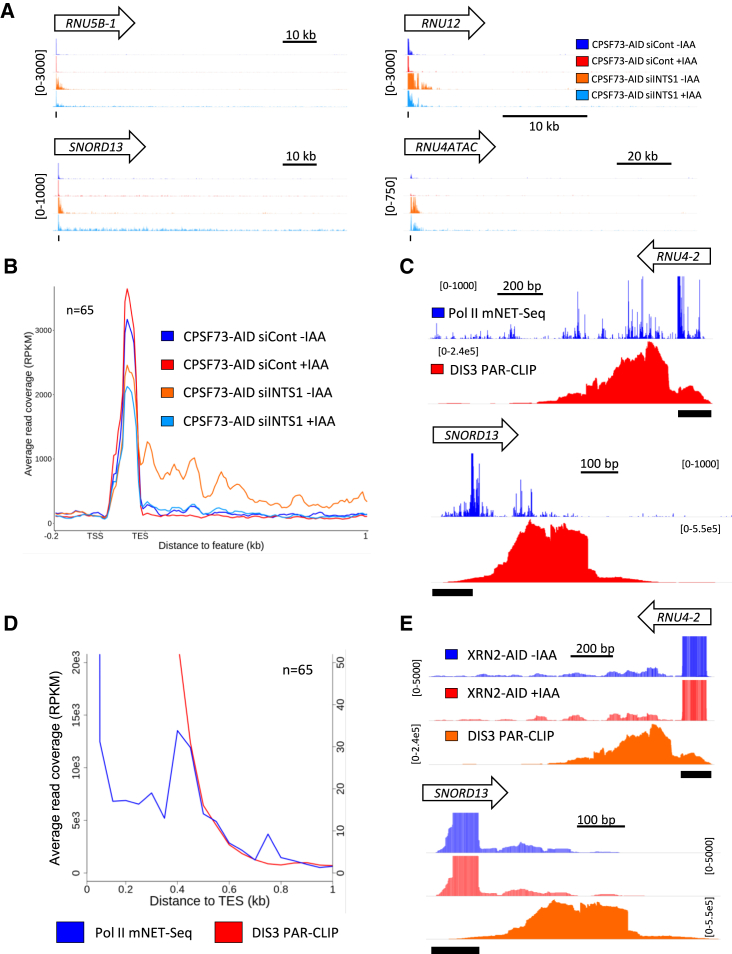
Redundant RNA Cleavage and Direct Termination Generate Some RNA 3′ Ends at snRNA Genes (A) Integrated Genomics Viewer (IGV) traces of *RNU5B-*1, *SNORD13*, *RNU12*, and *RNU4ATAC* (location indicated by black bar under each trace) from chromatin-associated RNA-seq performed in *CPSF73-AID* cells transfected with control or INTS1 siRNAs before treatment or not with auxin. The y axis shows RPKM. (B) Metaplot analysis of all snRNAs derived from samples used in (A). The y axis scales are RPKM. The –IAA samples (siCont and siINTS1) are the same as those shown in Figure 1F, as these data were obtained in the same experiment. (C) IGV tracks of *RNU4-2* and *SNORD13* (location indicated by black bar under each trace) comparing Pol II mNET-seq (blue) and published DIS3 PAR-CLIP (red) signals. mNET-seq signal is mapped to the 3′-most nucleotide of the read deriving from Pol II’s active center. “Spikes” of signal represent sites of greater Pol II occupancy. The y axis signals are RPKM. (D) Metaplot of mNET-seq versus DIS3 PAR-CLIP (Szczepińska et al., 2015) at all expressed snRNAs. The Pol II mNET-seq signal is very high at the final nucleotide of snRNAs because of the detection of their incorporation into spliceosomes (Nojima et al., 2015). Therefore, a zoomed view is provided to highlight nascent signals beyond this region. The y axes show RPKM and signals are in 50-bp bins. (E) IGV tracks of *RNU4-2* and *SNORD13* (location indicated by black bar under each trace) comparing DIS3 PAR-CLIP signals (Szczepińska et al., 2015) with nuclear RNA-seq obtained after auxin-dependent depletion of XRN2 (1-h depletion) (previously presented in Eaton et al., 2018). The y axis signals are RPKM.

We hypothesized that the exosome degrades snRNA precursors produced by non-Integrator cleavage activities or by termination itself. CPSF73 could provide alternative endoribonuclease activity and can be rapidly depleted from our previously described *CPSF73-AID* cells by using auxin (Eaton et al., 2020). Although termination of snRNA transcription is unaffected by CPSF73 elimination (Figures S2D and S2E; Eaton et al., 2020), its activity might only be relevant when Integrator is absent. We analyzed chromatin-associated RNA-seq performed in *CPSF73-AID* cells treated with control or INTS1 siRNAs before auxin addition. Co-depletion of CPSF73 with INTS1 enhances read-through at some (e.g., *SNORD13* and *RNU5B-1*) sno/snRNAs (Figure 3A). This finding implies auxiliary cleavage activities may be relevant when Integrator is depleted; however, this effect is not broadly apparent (Figure 3B). An interesting feature of the metagene profiles in Figure 3B is that CPSF73 depletion also reduces the effects of INTS1 loss, indicating some potential cross-talk between these nuclease activities. However, we previously noted reduced nascent transcription of many genes following CPSF73 depletion, with some evidence for this also seen downstream of snRNA genes (Figure S2F; Eaton et al., 2020).

Because CPSF73 activity does not generally explain Integrator-independent termination, we analyzed whether exosome-targeted snRNA precursors are generated by transcriptional termination. We mapped Pol II relative to DIS3 activity by using mammalian native elongating transcript sequencing (mNET-seq), which has single-nucleotide resolution (Nojima et al., 2015), and compared it to DIS3 occupancy uncovered by PAR-CLIP (Szczepińska et al., 2015). Although individual DIS3 and Pol II mNET-seq signals do not necessarily reveal their last possible position, a direct termination mechanism predicts little Pol II occupancy beyond the extremity of the DIS3 PAR-CLIP signal. In contrast, an endoribonuclease-based mechanism classically leads to downstream transcription and 5′→3′ degradation of a 3′ product (Eaton and West, 2020). Individual snRNAs show Pol II occupancy over DIS3-bound regions but much less coverage downstream of them, and a metagene analysis shows a close correlation between the decline of DIS3 and Pol II signals (Figures 3C and 3D). XRN2 elimination has no detectable impact on RNA surrounding these positions, suggesting that there are no substrates for its 5′→3′ exoribonuclease activity (Eaton et al., 2018 and Figure 3E). Although other 5′→3′ exonucleases cannot be completely ruled out, these data suggest that snRNA termination directly releases some DIS3 substrates.

### Exosome-Targeted Termination Occurs before snRNA Processing and Sometimes at T-Runs

**Figure 4:**
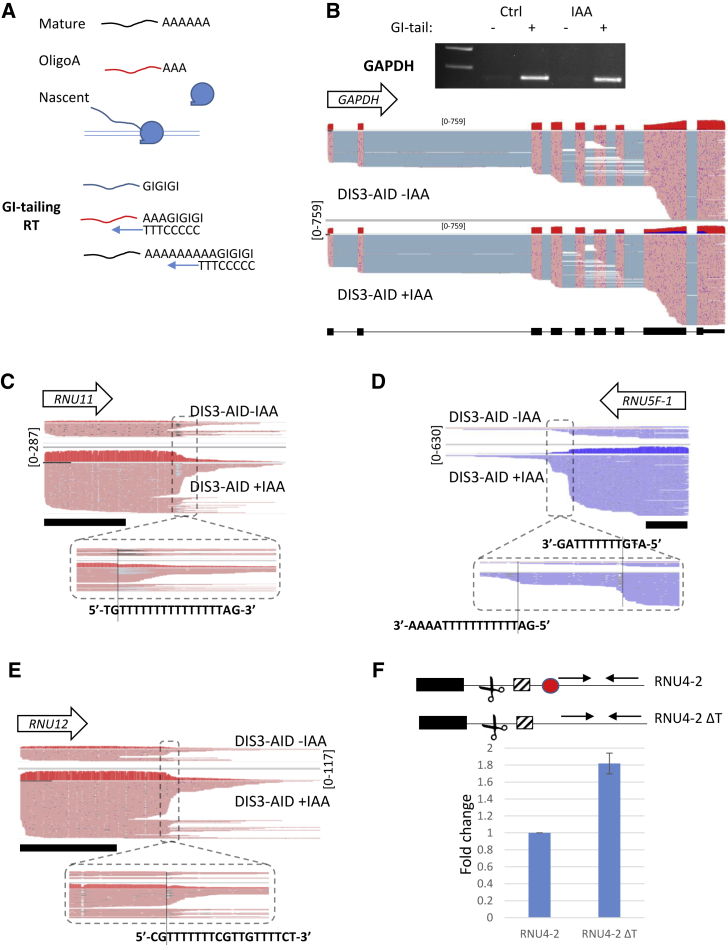
Analysis of Full-Length snRNA Precursors by Long-Read Sequencing (A) Schematic of long-read sequencing strategy. Nuclei contain mature polyadenylated RNAs with long poly(A) tails (black), terminated oligoadenylated species that might be exosome substrates (red), and unadenylated (blue) transcripts that, for example, might derive from Pol II. GI-tailing allows the sequencing of RNAs with pre-existing poly(A) (black) or oligoA (red) tails following an oligo-dC(T)_3_-primed cDNA synthesis step. (B) Agarose gel analysis of GAPDH transcripts isolated from *DIS3-AID* cells treated or not with auxin and/or GI-tailed, as indicated. Also shown is long-read sequencing tracks of GAPDH in GI-tailed samples. Each track shows coverage density at the top (red) scaled as transcripts per million (TPM) with individual reads displayed underneath. Individual reads show exons (pink) linked by introns removed by splicing. White space indicates unmapped regions between reads presumably truncated at their 5′/3′ ends. The blue signal coverage in auxin-treated samples derives from an annotated anti-sense RNA degraded by DIS3. (C–E) Long-read sequencing tracks of *RNU12*, *RNU11*, and *RNU5F-1* snRNAs from the GI-tailing experiment. Read density (TPM) and individual reads are shown. Regions of focused 3′ termini are indicated with a dashed box, and part of their primary sequence is shown. The black bar under each trace denotes the size and position of each mature snRNA as annotated. (F) qRT-PCR from HCT116 cells transfected with a plasmid expressing RNU4-2 or a derivative lacking a downstream T-tract (red circle). Primers were used to detect read-through RNA beyond the T-tract, as shown in the schematic. Graph shows fold increase relative to the unmodified construct after normalizing to GFP levels from a co-transfected control plasmid. n = 5. Error bars are SEM.

Allosteric/intrinsic termination in other systems often occurs at T-rich sequences in the non-template strand. To examine the terminal sequence(s) and upstream processing status of snRNA termination products, we used long-read nanopore sequencing on nuclear RNA from *DIS3-AID* cells treated or not with auxin. Long-read protocols normally detect polyadenylated RNA, but snRNA precursors may not have this modification. RNA was, therefore, GI-tailed to provide a common 3′ end to amplify (Figure 4A). To discriminate against RNA from within Pol II (which can also be GI-tailed *in vitro*), reverse -transcription was designed to enrich oligoadenylated 3′ ends, which are well-established features of exosome substrates (LaCava et al., 2005; West et al., 2006; Wyers et al., 2005). RNA-derived products were enriched by GI-tailing, and complete transcripts were recovered from GI-tailed samples, exemplified by GAPDH mRNA (Figure 4B).

Full-length snRNA precursors were also recovered, of which many could be terminated products (Figures 4C–4E). They are strongly stabilized by DIS3 loss and are not processed by Integrator, which data above anticipated by showing that DIS3 targeted precursors are poorly processed. The 3′ ends show a largely stochastic distribution, arguing against any sequence-defined endoribonucleolytic activity as predicted by our mNET-seq experiment and supporting termination as a possible source. Interestingly, precursors often stack over T-runs in the non-transcribed strand. In many systems, T-runs directly promote (e.g., Pol III) or facilitate (many prokaryotic RNA polymerases) transcriptional termination (Arimbasseri et al., 2014; Ray-Soni et al., 2016). 36% of the snRNAs that we analyzed showed evidence of termination at T > 4, a criteria selected because this is a minimal Pol III terminator (Table S1). Unlike for Pol III, Pol II termination is not inevitable at T-runs, as shown on *RNU5F-1* where reads extend beyond some T-stretches (Figure 4D). This has some analogy with bacterial intrinsic terminators, which culminate in a T-rich sequence but are variably efficient depending on other elements. Some snRNA precursors do not possess extensive T-runs but still exhibit stochastic 3′ termini that are not processed at the upstream canonical Integrator site (Figure S3). 3′ end mapping using an *Escherichia coli* poly(A) polymerase (EPAP) and short-read sequencing gave similar findings to these long-read data (Figures S4A–S4F). Although some 3′ ends detected in these experiments may still represent RNA cleavage sites, the absence of INTS11 cross-linking and the independence of such events from INTS1 argue against Integrator being responsible (Figure 2E andFigure S4G). Moreover, deletion of a single T-run from an *RNU4-2* reporter plasmid caused an enhancement of read-through, further arguing for a function of such elements in termination (Figure 4F).

Here, we identify a transcriptional termination mechanism at snRNAs that does not require canonical Integrator processing. Many such events appear to be allosteric/intrinsic in nature on the basis that 5′→3′ exonuclease activity (XRN2) is not implicated. We suggest that Pol II is generally prone to terminate stochastically downstream of snRNAs, with this activity focused by T-runs. T-runs are intrinsic terminators of other RNA polymerases and of purified Pol II *in vitro* (Dedrick et al., 1987), but we do not rule out the involvement of proteins. In budding yeast, termination of snRNA transcription uses the Sen1 helicase, and mapping of its terminated transcripts reveals U-rich regions at the 3′ ends that could aid this process (Schaughency et al., 2014). Finally, Integrator is broadly involved in early Pol II termination, including at promoter proximal and enhancer regions (Beckedorff et al., 2020; Lai et al., 2015; Nojima et al., 2018; Stadelmayer et al., 2014). In those cases, its depletion does not prevent termination to the extent that CPSF73 elimination does at protein-coding genes, which might be due to alternative pathways akin to those identified here.

## STAR★Methods

### Key Resources Table

**Table:** 

REAGENT or RESOURCE	SOURCE	IDENTIFIER
**Antibodies**		

INTS1	Bethyl	Cat# A300-361A; RRID: AB_2127258
INTS11	Abbexa	abx005038
Tubulin	Abcam	Cat# Ab7291; RRID: AB_2241126
SPT5	Santa Cruz	Cat# sc-133217; RRID: AB_2196394
RNA pol II (ChIP-seq)	Abcam	Cat# Ab817; RRID: AB_306327
RNA pol II (mNET-seq)	MBL	MABI601 (discontinued)

**Chemicals, Peptides, and Recombinant Proteins**		

JetPrime	VWR	114-07
Lipofectamine RNAiMAX	Fisher Scientific	13778075
Asunaprevir		
Auxin	Sigma	I2886
Hygromycin	Life Technologies	10687010
Neomycin	Sigma	345810
LUNA qPCR mastermix	NEB	M3003
Protoscript II	NEB	M0368
TURBO DNase	Fisher Scientific	AM2238
Benzonase	Sigma	E1014
Tri-reagent	Fisher Scientific	AM9738
Sheep anti-mouse dynabeads	Thermo fisher	11201D
Doxycycline	Sigma	D3447
Triptolide	Sigma	T3652
Asunaprevir	Cambridge Biosciences	CAY20835-1

**Critical Commercial Assays**		

RNA-seq library preparation kit	Illumina	20020597
ChIP-seq Sample preparation kit	Cell Signaling Technologies	9003S
ChIP-seq Library preparation kit	NEB	E7103
EPAP experiment RNA-seq library preparation kit	Lexogen	SKU: 016.24
Long read sequencing kit	Oxford Nanopore	SQK-PCS109
Long read sequencing flow cell	Oxford Nanopore	SQK-PCS109
Long read flow cell wash kit	Oxford Nanopore	EXP-WSH002
Poly(A) tail length assay kit (GI tailing)	Thermo Fisher	764551KT
Poly(A) tailing kit (EPAP treatment)	Thermo FIsher	AM1350
Plasmid miniprep kit	QIAGEN	27106
RNA microprep kit	Zymo	R1050
mNET-seq library prep kit	NEB	E7330
Ribozero rRNA depletion kit	Illumina	Discontinued but replaced by 20037135

**Deposited Data**		

INTS11-SMASh nuclear RNA-seq; DIS3-AID cells long-read sequencing; DIS3-AID cells EPAP RNA-seq; Pol II ChIP-seq in CPSF73-AID cells; mNET-seq analysis of Pol II occupancy	This paper	Gene expression omnibus: GSE150238
CPSF73-AID treated with siCont, siINTS1, +/−auxin	This paper and Eaton et al., 2020	Gene expression omnibus: GSE150238; GSE137727
Pol II ChIP-seq control versus INTS11 RNAi	Stadelmayer et al., 2014	Gene expression omnibus: GSE60586
DIS3 PAR-CLIP	Szczepińska et al., 2015	Gene expression omnibus: GSE64332
DIS3-AID cell nuclear RNA-seq ± auxin	Davidson et al., 2019	Gene expression omnibus: GSE120574
XRN2-AID cell nuclear RNA-seq ± auxin	Eaton et al., 2018	Gene expression omnibus: GSE109003
INTS11 eCLIP data	Barra et al., 2020	Gene expression omnibus: GSE148755
Raw western blot/agarose gels	Mendeley Data	

**Experimental Models: Cell Lines**		

HCT116 *INTS11-SMASh*	This paper	N/A
HCT116 *DIS3-AID*	Davidson et al., 2019	N/A
HCT116 *CPSF73-AID*	Eaton et al., 2020	N/A
HCT116 *XRN2-AID*	Eaton et al., 2018	N/A

**Oligonucleotides**		

See Table S2	IDT	Table S2, this paper

**Recombinant DNA**		

pCS6-YFP-SMASh	Addgene	68853
px330	Addgene	42230
P2A-NEO/HYG-SV40 PAS sequences	Eaton et al., 2018	N/A
AID sequence	Davidson et al., 2019	N/A
INTS11-SMASh homology arms	This paper	Table S2
RNU4-2 plasmid	This paper	Table S2

**Software and Algorithms**		

Bamtools v2.4.0	Barnett et al., 2011	N/A
BEDtools v2.26.0	Quinlan and Hall, 2010	N/A
Cutadapt v1.15;2.9	Martin, 2011	N/A
Deeptools v3.0.2;3.4.2	Ramírez et al., 2014	N/A
FastQC v0.11.9	https://www.bioinformatics.babraham.ac.uk/projects/fastqc/	N/A
featureCounts v2.0.0	Liao et al., 2013, 2014	N/A
Guppy v2.3.7	Oxford Nanopore Technologies LTD	N/A
Hisat2 v.2.1.0	Kim et al., 2015	N/A
IGV v2.8.2	Robinson et al., 2011	N/A
minimap2 v2.16-r934-dirty	Li, 2018	N/A
MultiQC v1.8	Ewels et al., 2016	N/A
Porechop v0.2.4	https://github.com/rrwick/Porechop	N/A
Pychopper v2.0.2	https://github.com/nanoporetech/pychopper	N/A
R v3.6.1	https://cran.r-project.org/	N/A
SAMTools 1.4.1;1.10	Li et al., 2009	N/A
Trim_Galore v0.4.4;0.6.5	http://www.bioinformatics.babraham.ac.uk/projects/trim_galore/	N/A

### Resource Availability

#### Lead contact

Further information and requests for resources and reagents should be directed to and will be fulfilled by the Lead Contact, Steven West (s.west@exeter.ac.uk).

#### Materials availability

New reagents generated in this study are available via the lead contact.

#### Data and code availability

The accession number for the new sequencing datasets reported in this paper is Gene Expression Omnibus: GSE150238.

### Experimental Model and Subject Details

This study employed human HCT116 cell lines (male-derived) and derivatives thereof. These were maintained at 37°C, 5% CO_2_ and grown in dulbeccos modified eagle medium (DMEM) containing 10% fetal bovine serum. *INTS11-SMASh* cells were obtained by transfecting a subconfluent 6-well dish with 1ug px330, containing the INTS11 guide RNA sequence, and 1ug each of SMASh neomycin and hygromycin repair constructs using Jetprime. Media was refreshed after 16 hr and after a further 48 hr cells were expanded to a 100mm dish containing 30ug/ml hygromycin and 800ug/ml G418. ∼10-14 days later, single colonies were picked and expanded for genomic PCR confirmation of *INTS11* modification. *DIS3-AID* cells are described in detail elsewhere as are the nucleotide sequences of P2A, Neomycin, Hygromycin and the SV40 PAS included in the HDR templates (Davidson et al., 2019; Eaton et al., 2018).

### Method Details

#### INTS11-SMASh cell line generation and other cloning

*INTS11* homology arms were synthesized by Integrated DNA Technologies and inserted into a pUC-based plasmid using Gibson Assembly. The SMASh tag was PCR isolated from pCS6-YFP-SMASh and inserted into our previously described Hygromycin/Neomycin selection vectors (Eaton et al., 2018), from which an AID tag had been removed by PCR. The resulting insert containing SMASh, a P2A cleavage sequence, the drug marker and an SV40 PAS was isolated by PCR and cloned into the INTS11 homology arm vector, that had been linearized at the stop codon by PCR, using Gibson assembly. The INTS11 guide RNA was designed using Benchling and cloned into px330 digested with BbsI. RNU4-2 sequences were isolated from HCT116 genomic DNA and inserted into a plasmid prepared by PCR amplification of a pcDNA5 FRT/TO plasmid containing the human beta-globin gene. The resulting plasmid replaced beta-globin and the upstream CMV promoter with the RNU4-2 sequence (see Table S2 for primers).

#### Cell culture and experimental manipulation

For INTS11 depletion ASN was added at 3uM final concentration for 48 hr. For INTS1/EXOSC3 RNAi, 6 well dishes were transfected with control or INTS1 siRNA using Lipofectamine RNAiMAX following the manufacturers protocol. The siRNA transfection was repeated after 24 hr and RNA was isolated 48 hr after that. For DIS3-AID depletion, auxin was added to a final concentration of 500uM. For CPSF73-AID depletion 1ug/ml doxycycline was added for 18 hr before treatment with auxin. ActD and triptolide were used at concentrations of 5ug/ml and 1uM triptolide respectively.

#### RNA isolation for qRT-PCR

This was generally performed in a 24-well dish using Tri-reagent following the manufacturer guidelines. Isolated RNA was treated with Turbo DNase for 1 hr at 37 degrees before phenol chloroform extraction and ethanol precipitation. 1ug of RNA was reverse transcribed using Protoscript II and 1/50^th^ of the cDNA was used for real-time PCR which was performed on a QIAGEN Rotorgene using LUNA qPCR mastermix.

#### Nuclear RNA-seq

Nuclei were extracted from a sub-confluent 100mm dish of *INTS11-SMASh* cells after 0 or 48 h of asunaprevir treatment using 4ml HLB (10 mM Tris pH5.5, 10 mM NaCl, 2.5 mM MgCl2, 0.5% NP40) underlayerd with 1ml HLB with 10% sucrose. After spinning for 5 min (500xg) the nuclear pellet was resuspended in 1ml of Tri-reagent and RNA isolated as described for qRT-PCR above. RNA quantity was determined using a Nanodrop 2000 spectrophotometer (Thermo) and its quality was assessed using a Tapestation (Agilent). 1 μg was rRNA-depleted and libraries were generated using TruSeq Stranded Total RNA Library Prep Kit.

#### Chromatin-associated and nucleoplasmic RNA isolation and sequencing

Nuclei were isolated as above then re-suspended in 100ul NUN1 (20mM Tris-HCl at pH 7.9, 75 mM NaCl, 0.5 mM EDTA, 50% glycerol, 0.85 mM DTT) and incubated for 5 min on ice before the addition of 1 mL of NUN2 buffer (20 mM HEPES at pH 7.6, 1 mM DTT, 7.5 mM MgCl2, 0.2 mM EDTA. 0.3 M NaCl, 1 M urea, 1% NP40). Cells were incubated on ice for 10 mins and shook at 2-3min intervals. After spinning (13000 rpm, 10 mins), nucleoplasmic RNA was phenol chloroform extracted from the supernatant and concentrated by ethanol precipitation. The pellet (containing chromatin-associated RNA) was re-suspended in Trizol and incubated for ∼30 mins at 37 degrees. RNA was then isolated as per the manufacturers guidelines. 1ug was prepared and used for RNA sequencing as described for nuclear RNA. Chromatin-associated RNA-seq (Figures 1F, 3A, and 3B) was performed in *CPSF73-AID* cells and the control siRNA samples -/+IAA were previously analyzed for protein-coding termination defects (Eaton et al., 2020). INTS1 siRNA samples are first described in the present paper.

#### GI-tailing and nanopore sequencing

Nuclear RNA extracted from *DIS3-AID* cells treated, or not, with IAA. This was first treated with RiboZero (Illumina) and GI-tailed using the poly(A) tail-length assay kit. The final RNA quantity and average length were determined using an HS Tapestation (Agilent). 50 ng of input RNA was reverse transcribed into cDNA using a modified VNP primer, downstream steps were performed using the cDNA-PCR Sequencing Kit (SQK-PCS109) according to the protocol. For each library, we loaded 100 fmol sequentially on a MK 1 R9 flow cell and sequenced for ∼4 h per library on a MinION device. The flow cell was washed between loading and sequencing of each library using a Wash Kit according to the user manual.

#### EPAP tagging and sequencing

1ug of chromatin-associated or nucleoplasmic RNA was treated or not with EPAP (30 min, 30 degrees) then depleted of rRNA. Sequencing libraries were made using the QUANT-seq REV kit.

#### mNET-seq

The mNET-seq library protocol was performed as described by Nojima et al. (2016) with the following modifications. Two sub-confluent 150mm dishes of cells were used per sample and chromatin pellets isolated as described above. The chromatin pellet was digested with micrococcal nuclease for 2 mins at 37 degrees. After inactivating the nuclease (25mM EGTA), the supernatant was incubated for 1 hr with magnetic beads (Sheep anti-mouse dynabeads) that had been preincubated for 2 hr with anti-Pol II. Immunoprecipitated RNA was 5′ phosphorylated before purification of fragments between 17-200nt using a Quick-RNA microprep kit (Zymo). Libraries were prepared for sequencing using the NEB Next Small RNA sequencing kit.

#### ChIP-sequencing

One 100mm *CPSF73-AID* cells were used per condition. ChIP was performed with the Simple ChIP enzymatic chromatin IP kit exactly as described in the product guidelines with one exception. Antibody (5ug Pol II, 8WG16) was coupled to sheep anti-mouse dynabeads instead of the beads included in the kit. Sequencing libraries were generated with the NEBNext® Ultra II DNA Library Prep Kit.

### Bioinformatics

#### Illumina-Sequenced Short-Read Alignment

The quality of demultiplexed raw 50 bp fastq reads was assessed using a combination of FastQC and MultiQC, before adaptor trimming using Trim_Galore (wrapper for Cutadapt) with default settings. Adaptor trimmed reads passing both length and quality cut-offs were then mapped to GRCh38 (Ensembl) using Hisat2 (Kim et al., 2015). Unmapped and multi-mapped and reads with a MAPQ score < 30 were discarded using SAMtools (Li et al., 2009).

#### EPAP Read Processing and Alignment

Adaptor sequences were first removed from raw 50 bp fastq EPAP reads using Trim_Galore with default setting. Trimmed reads were screened using FastQC and MultiQC to confirm adaptor removal before trimming a second time using Trim_Galore, this time to remove long, non-encoded 3′ poly(A) and poly(T) sequences. Following this, reads were mapped to GRCh38 using Hisat2 (Kim et al., 2015), discarding unmapped, multi-mapped and low MAPRQ scored (< 30) reads using SAMTools (Li et al., 2009).

#### ONT Basecalling and Read Alignment

The fast5 sequences were base-called and converted to a fastq file format using Guppy before being passed to pychopper to extract full-length reads and orientate them by strand. Adaptor sequences were then removed from full-length reads using porechop and mapped to GRCh38 using minimap2 (Li, 2018), with the following settings:

-a -k 15 -w 5–splice -g 2000 -G 200k -A 1 -B 2 -O 2,32 -E 1,0 -C 9 -z 200 -u f–junc-bonus = 9
–splice-flank = yes–no-long-join–secondary = no

Aligned reads were then filtered to remove unmapped, multi-mapped and low MAPQ (< 30) scored reads using the SAMTools suite (Li et al., 2009).

#### mNET-seq mapping

The mNET-seq traces used single-nucleotide resolution BAM files corresponding to the 3′ end of the RNA fragment (Nojima et al., 2015).

#### Generating Normalized Read Coverage BigWigs

For IGV visualization and metagene analysis, libraries were merged with Bamtools (Barnett et al., 2011). Normalized read coverage files of both short-read (Illumina) and long-reads (ONT) were produced using the deepTools at a single nucleotide resolution of sense and antisense separated strands (Ramírez et al., 2014). Illumina reads were normalized to Reads Per Kilobase of transcript, per Million mapped reads (RPKM), whereas ONT reads were normalized to Transcripts-Per-Million (TPM), ignoring reads with a MAPQ score < 30.

#### Metagene Analysis

For snRNA metagene analysis, expressed snRNA genes (> 5 reads in untreated INTS11 cells) were selected from 157 ensembl annotated snRNA genes present in https://rnacentral.org/ and a window extending 1 kb downstream of each TES was then calculated and the snRNA gene body was scaled to 200bp. To prevent counting of ambiguous reads, snRNA genes near annotated genes (< 1kb downstream of their TES) were discarded using the BEDtools suite (Quinlan and Hall, 2010). Pol III genes were removed. Metagene profiles were then calculated using RPKM normalized read coverage with further graphical processing performed in the R environment.

For CPSF73-AID RNA Pol II ChIP metagene analysis all annotated snRNA genes were selected and a window of 1 kb downstream of the TES was incorporated. Due to differences in signal coverage between the CPSF73-AID samples, the auxin treated profile was scaled to the control profile using the mean signal difference over the gene body (scaled to 200 bp). Further graphical processing of metagene plots was performed in the R environment.

### Quantification and Statistical Analysis

qRT-PCR was quantitated using the comparative quantitation function associated with the QIAGEN Rotorgene instrument. Values were first normalized to a loading control (stated in the relevant figure legend) and then samples were compared by quantitating the experimental values relative to the control condition (given the value of 1 by the software). Bars show the average of at least three replicates (exact n provided in figure legends) and error bars show the standard deviation of the mean.

## Supplemental Information

Document S1. Figures S1–S4 and Table S1 and S2

Document S2. Article plus Supplemental Information
