# Supplementary material for: Integrator-Dependent and Allosteric/Intrinsic Mechanisms Ensure Efficient Termination of snRNA Transcription
Source: Cell Rep. 2020 Oct 27;33(4):108319. doi: 10.1016/j.celrep.2020.108319 (PMC7610016; doi:10.1016/j.celrep.2020.108319)
Supplement: Document S1. Figures S1–S4 and Table S1 and S2 [file mmc1.pdf]

Cell Reports, Volume *33*

Supplemental Information

Integrator-Dependent and Allosteric/Intrinsic Mechanisms Ensure Efficient Termination of snRNA Transcription

Lee Davidson, Laura Francis, Joshua D. Eaton, and Steven West

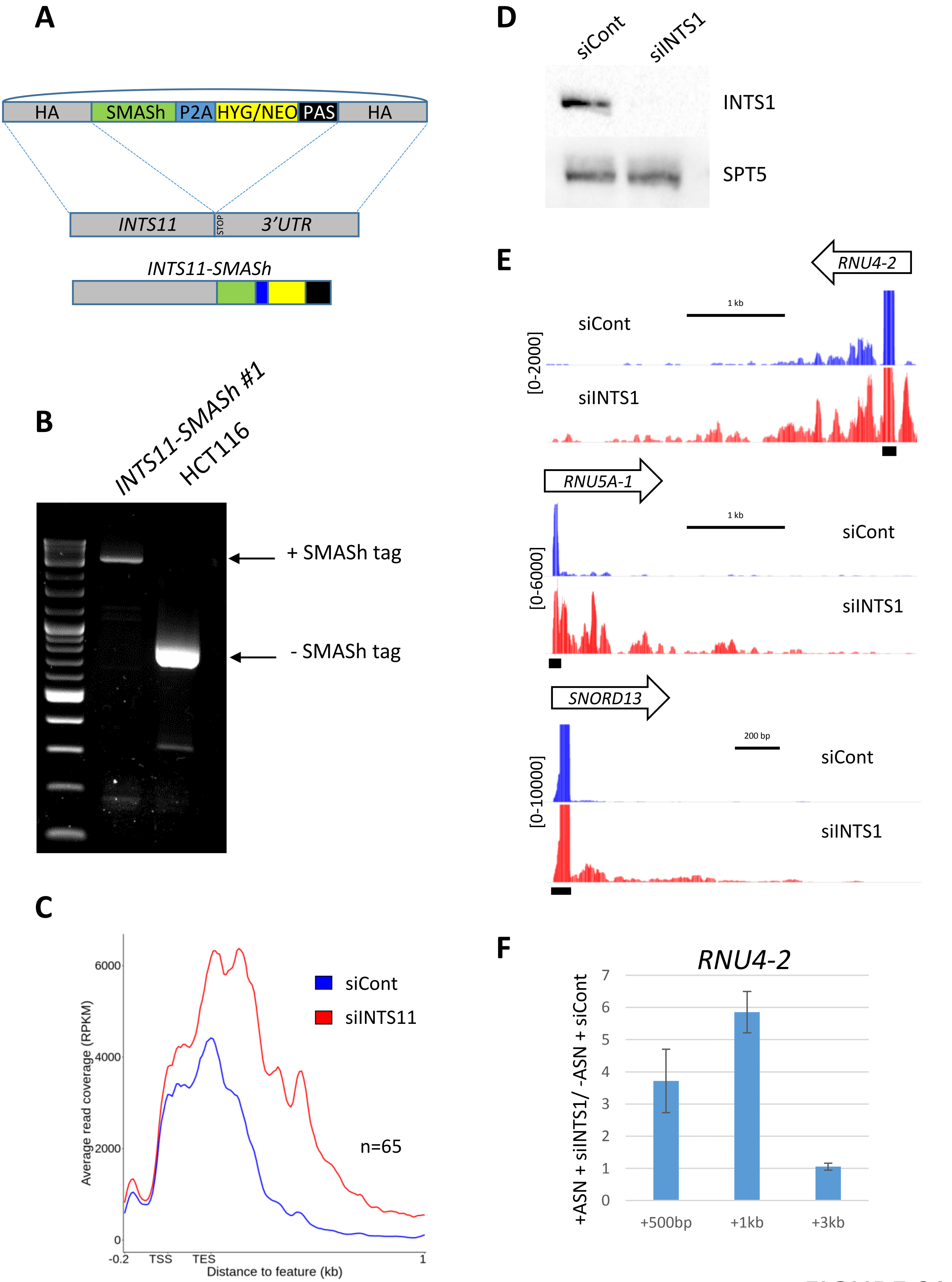

A
HA
SMASh
P2A
HYG/NEO
PAS
HA
INTS11
STOP
3'UTR
INTS11-SMASh
B
INTS11-SMASh #1
HCT116
+ SMASh tag
- SMASh tag
C
Average read coverage (RPKM)
6000
4000
2000
0
-0.2
TSS
TES
1
Distance to feature (kb)
siCont
siINTS11
n=65
D
siCont
siINTS1
INTS1
SPT5
E
RNU4-2
1 kb
siCont
[0-2000]
siINTS1
RNU5A-1
1 kb
siCont
[0-6000]
siINTS1
SNORD13
200 bp
siCont
[0-10000]
siINTS1
F
RNU4-2
+ASN + siINTS1/ -ASN + siCont
7
6
5
4
3
2
1
0
+500bp
+1kb
+3kb

FIGURE S1

FIGURE S1. Integrator depletion delays but does not abolish termination of snRNA transcription, Related to figure 1

A. Schematic of the strategy for inserting the SMASh tag into *INTS11*. The homology-directed repair plasmid is shown with various components coloured and labelled. SMASh is followed by a P2A site, neomycin/hygromycin (Neo/Hyg) resistance markers and an SV40 PAS. While transcribed as a single mRNA, P2A cleavage during translation ensures two separate proteins: INTS11-SMASh and Neo/Hyg.

B. PCR verification of the clone taken forward for study. Primers were used outside of the homology arms such that an upshift in product size would be observed as a result of cassette integration. As can be seen, the modified cell line shows this upshift product with a complete loss of the product indicative of no modification, which is present only in unmodified cells.

C. Metaplot of Pol II ChIP density over-expressed snRNAs in HeLa cells treated with control or INTS11-specific siRNAs. Signal is RPKM. Data are from (Stadelmayer et al., 2014)

D. Western blot of INTS1 levels in *DIS3-AID* cells transfected with control or INTS1-specific siRNAs. SPT5 is also probed for as a loading control.

E. Integrated genome viewer (IGV) snapshots of *RNU4-2, RNU5A-1* and SNORD13 in chromatin-associated RNA-seq obtained after transfection with control or INTS1-specific siRNAs. The signal upstream of *RNU4-2* in the RNAi scenario is read-through from *RNU4-1* positioned shortly upstream. Signal is RPKM. *RNU4-2*, 141bp; *RNU5A-1*,116bp and *SNORD13*, 104bp are shown as a black bar under each trace.

F. qRT-PCR analysis of *INTS11-SMASh* cells transfected with control or INTS1-specific siRNAs before treatment or not with ASN. Levels of RNU4-2 extended read-through was assayed. Position of each amplicon beyond the 3' end processing site is indicated under the graph. Quantitation is relative to non-ASN treated siCont transfected samples after normalising to spliced actin levels. n=3, error bars are SEM.

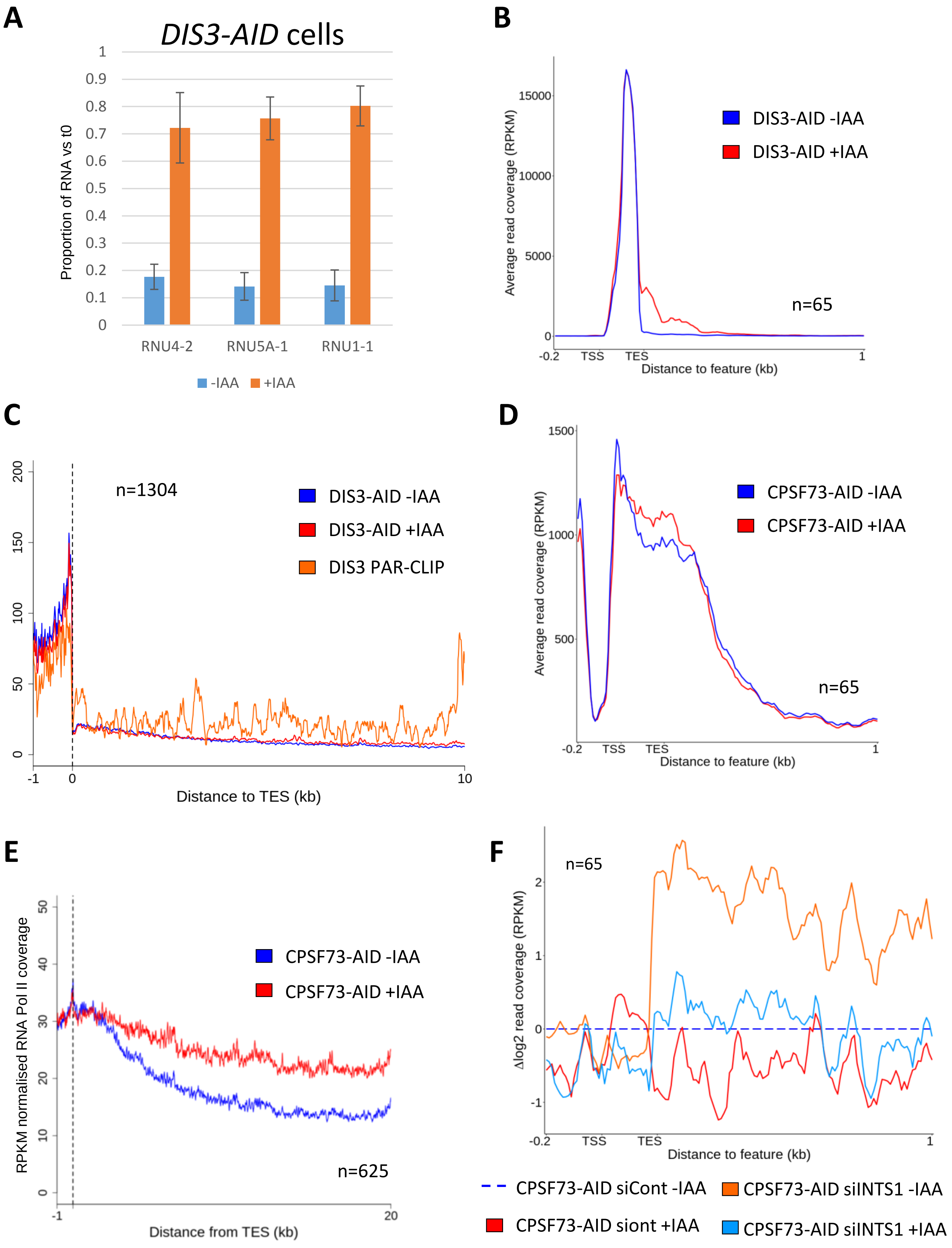
A
DIS3-AID cells
Proportion of RNA vs t0
1
0.9
0.8
0.7
0.6
0.5
0.4
0.3
0.2
0.1
0
RNU4-2
RNU5A-1
RNU1-1
-IAA
+IAA
B
Average read coverage (RPKM)
15000
10000
5000
0
DIS3-AID -IAA
DIS3-AID +IAA
n=65
-0.2
TSS
TES
1
Distance to feature (kb)
C
200
150
100
50
0
n=1304
DIS3-AID -IAA
DIS3-AID +IAA
DIS3 PAR-CLIP
-1
0
10
Distance to TES (kb)
D
1500
1000
500
Average read coverage (RPKM)
CPSF73-AID -IAA
CPSF73-AID +IAA
n=65
-0.2
TSS
TES
1
Distance to feature (kb)
E
RPKM normalised RNA Pol II coverage
50
40
30
20
10
0
CPSF73-AID -IAA
CPSF73-AID +IAA
n=625
-1
20
Distance from TES (kb)
F
n=65
Δlog2 read coverage (RPKM)
2
1
0
-1
-0.2
TSS
TES
1
Distance to feature (kb)
CPSF73-AID siCont -IAA
CPSF73-AID siINTS1 -IAA
CPSF73-AID siont +IAA
CPSF73-AID siINTS1 +IAA

FIGURE S2

FIGURE S2. DIS3 and CPSF73 effects on snRNA processing and read-through RNA, Related to figures 2 and 3

A. qRT-PCR analysis of snRNA precursor stability in *DIS3-AID* cells treated or not with auxin. Quantitation shows the proportion of precursor remaining after 15 mins triptolide treatment versus the respective untreated condition (t0). n=3, error bars are SEM.

B. Meta-analysis of nuclear RNA-seq data obtained in *DIS3-AID* cells treated or not with auxin (1 hr) to assay signal over all expressed snRNA genes (Davidson et al., 2019). Y-axis shows RPKM.

C. Metaplot of all expressed protein-coding genes separated from neighbouring genes by at least 10kb. The region downstream of the PAS (TES) is shown. This shows DIS3-PAR clip signal and reads obtained from our previously published RNA-seq performed in *DIS3-AID* cells treated or not with auxin (1 hr) (Davidson et al., 2019; Szczepinska et al., 2015). Y-axis scale is RPKM. It is clear that DIS3 does not occupy this region and its depletion does not affect read-through RNA levels.

D. Metaplot of snRNA genes generated from the Pol II ChIP-seq performed in *CPSF73-AID* cells treated or not with auxin (3hr). Y-axis scale is RPKM.

E. Metaplot of protein-coding genes that do not contain another gene within 20kb of their TES, generated from the Pol II ChIP-seq performed in *CPSF73-AID* cells treated or not with auxin (3hr). Signal was normalised to gene body levels in each case. Y-axis scale is RPKM. This shows that the negative result in B is not due to incomplete CPSF73 elimination or another experimental failure.

F. Δlog2 representation of the metagene present in main text Figure 3B. This demonstrates the reduced level of 3' flanking RNA in samples depleted of CPSF73 (red line). This likely contributes to the apparent reduced impact of INTS1 depletion in this scenario (light blue trace).

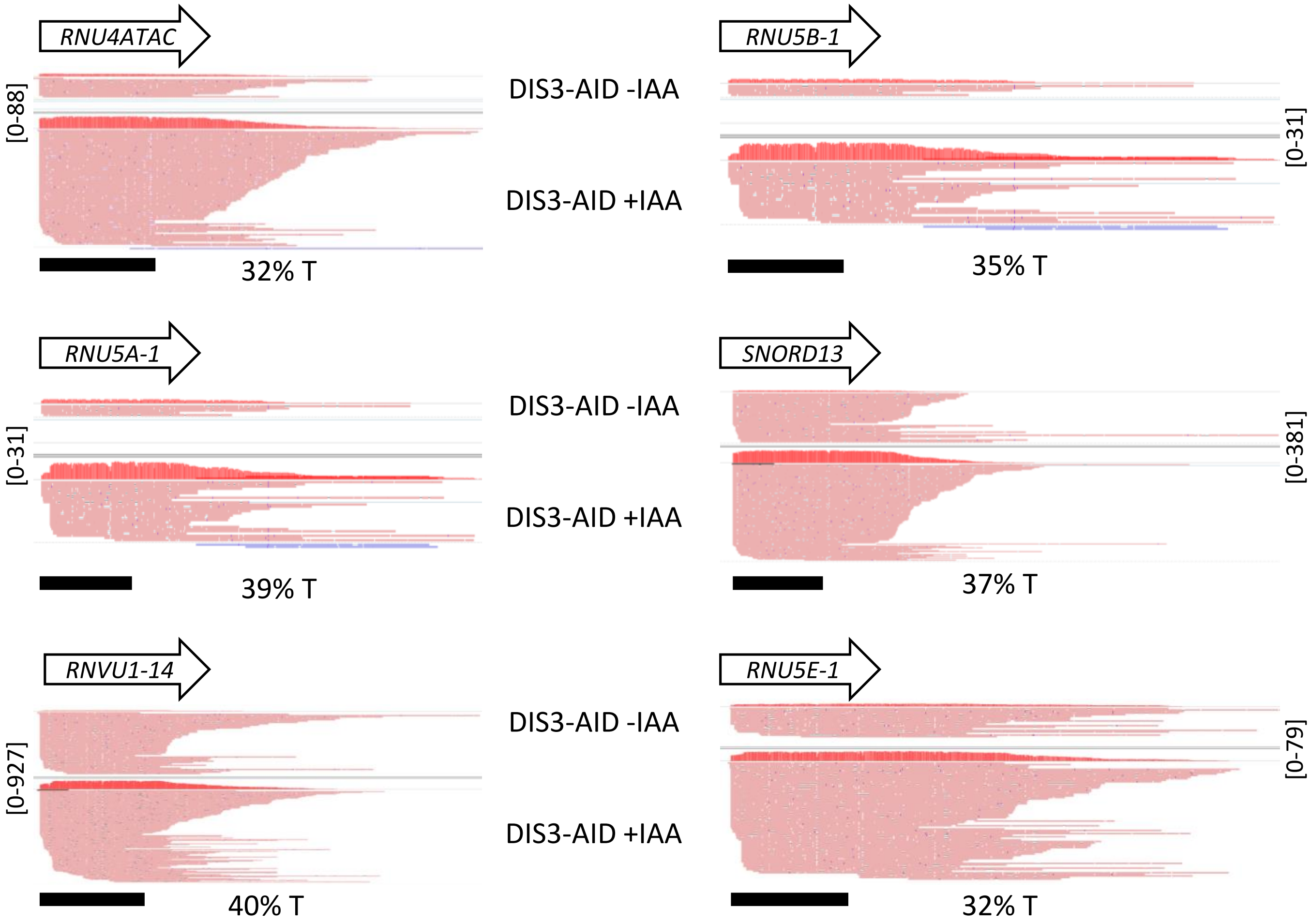

FIGURE S3. Examples of snRNA nanopore sequence traces, Related to figure 4

IGV tracks showing long-read coverage over a collection of sn/snoRNA genes (as labelled) in samples from *DIS3-AID* -cells treated or not with auxin (3hr) to complement those in Figure 4. In all cases, there are stochastic 3' ends that are stabilised by exosome loss. These do not always stack over specific regions though terminator regions have high T content (indicated). By contrast, the 5' ends of most reads map to the expected transcriptional start site regions. Note that some snRNAs show short truncations at their 5' ends. The possibility that these show 5' degradation intermediates cannot be distinguished from the possibility that they represent incomplete cDNA synthesis/5' degradation during RNA isolation. Y-axis scales are TPM. Genes are labelled and their precise position is indicated by the black bars which provide length scale. *RNU4ATAC*, 127bp; *RNU5B-1*, 116bp; *RNU5A-1*, 116bp; *SNORD13*, 104bp; *RNVU1-14*, 164bp; *RNU5E-1*, 120bp.

FIGURE S3

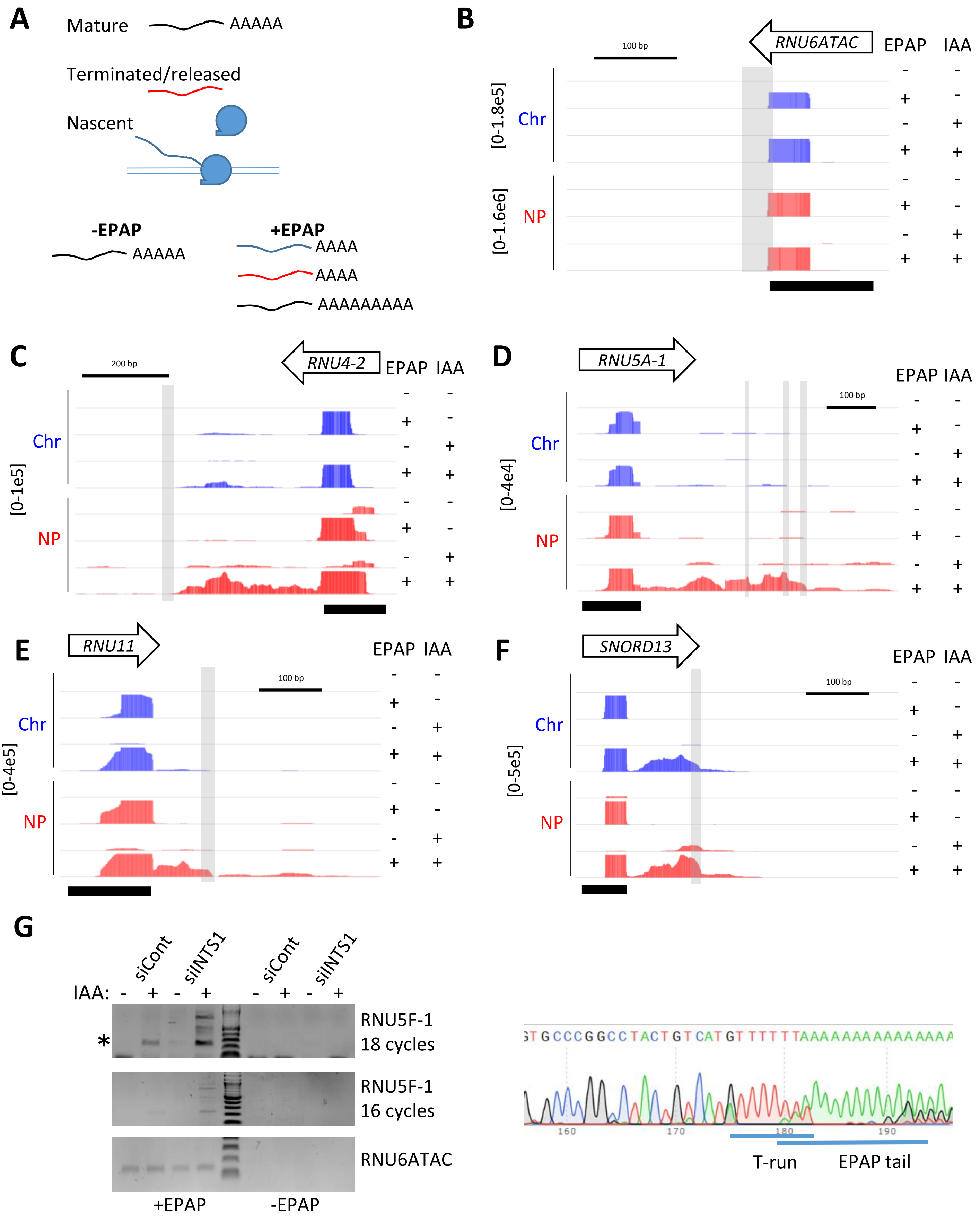
A
Mature
AAAAA
Terminated/released
Nascent
-EPAP
AAAAA
+EPAP
AAAA
AAAA
AAAAAAAAA
B
100 bp
RNU6ATAC
EPAP
IAA
[0-1.8e5]
Chr
[0-1.6e6]
NP
C
200 bp
RNU4-2
EPAP IAA
[0-1e5]
Chr
NP
D
RNU5A-1
100 bp
EPAP IAA
[0-4e4]
Chr
NP
E
RNU11
100 bp
EPAP IAA
[0-4e5]
Chr
NP
F
SNORD13
100 bp
EPAP IAA
[0-5e5]
Chr
NP
G
siCont
siINTS1
siCont
siINTS1
IAA:
*
RNU5F-1
18 cycles
RNU5F-1
16 cycles
RNU6ATAC
+EPAP
-EPAP
GTGCCCGGCCTACTGTCATGTTTTTAAAAAAAAAAAAAA
160
170
180
190
T-run
EPAP tail

FIGURE S4

FIGURE S4. Evidence of termination at T-runs obtained via in vitro polyadenylation and sequencing of 3' ends, Related to figure 4

A. Schematic of an orthogonal 3' end mapping method. Nuclear RNA is treated or not with *E.coli* poly(A) polymerase (EPAP) before 3' end mapping by oligodT primed RNA-seq. Without EPAP, only RNAs with an existing poly(A) tail (black) can be mapped. However, EPAP treatment detects transcripts that are not normally polyadenylated. These might include transcripts isolated from engaged Pol II (blue; chromatin-associated) or other transcripts released from chromatin (red; nucleoplasmic).

B. IGV track for the Pol III-transcribed U6 ATAC gene. Tracks show chromatin (blue) and nucleoplasmic (red) signals deriving from RNAs isolated from *DIS3-AID* cells treated or not with auxin (2hr). EPAP and auxin treatments are indicated beside the traces. U6 ATAC is not normally polyadenylated and is only detected in samples treated with EPAP demonstrating the capacity of this method to differentiate polyadenylated and non-polyadenylated 3' ends. Y-axis scale is RPKM. Gene is labelled and its position on the trace is noted by the black bars which provide length scale. *RNU6ATAC* is 126bp. The shaded region denotes the T-run terminator

C-F. Examples of sn and representative snoRNAs. Labelling is as per B. Transcripts are enriched by EPAP treatment and more abundant when auxin is used demonstrating their normally unadenylated status and susceptibility to DIS3. Signal is more abundant in nucleoplasmic RNA consistent with release of exosome substrates after termination of transcription. However, stabilised reads are also evident in the chromatin-associated fraction consistent with their production by termination (i.e. that they are nascent). We do not know whether there is any subsequent nuclear export/reimport of these species. Note that there is more enrichment of reads further into the 3' flank than for long-read sequencing. This is probably because PCR amplification of longer reads will generally be more biased in favour of shorter cDNAs compared to this short-read approach. Y-axis scale is RPKM. Genes are labelled and their precise position is indicated by the black bars which provide length scale. *RNU4-2*, 141bp; *RNU5A-1*, 115bp; *SNORD13*, 104bp, *RNU12*, 150bp. Shaded areas indicateT-runs.

G. Analysis of 3' terminated precursors from *RNU5F-1* recovered from *DIS3-AID* cells transfected with control or INTS1 siRNAs before treatment or not with auxin. Before PCR amplification, samples were treated or not with EPAP as indicated. Termination products are seen following depletion of DIS3 but are most enriched when DIS3 and INTS1 are co-depleted. The co-depletion reveals additional downstream termination products showing that upstream sites are not sufficient for complete termination. Signals are dependent on EPAP demonstrating that they are genuine 3' ends. Two panels are shown for RNU5F-1 representing different cycle numbers. U6ATAC is a loading control and is detected via the EPAP polyadenylation of its 3' end which is known to be formed at a T-run. The sequencing trace of the asterisked PCR product is shown and confirms its termination at a T-run. Interestingly, the EPAP tail is not evident prior to the 4th T in the tract indicating that a minimum of 4 T's are required to release the RNA at this position.

Ensembl ID	Gene ID	Ensembl ID	Gene ID
ENSG00000199347	RNU5E-1	ENSG00000212413	RNU11-3P
ENSG00000206652	RNU1-1	ENSG00000201616	RNU1-91P
ENSG00000207389	RNU1-4	ENSG00000221439	RNU4ATAC16P
ENSG00000207005	RNU1-2	ENSG00000201435	RNU4-24P
ENSG00000201609	RNU4-28P	ENSG00000202538	RNU4-2
ENSG00000274978	RNU11	ENSG00000200795	RNU4-1
ENSG00000199377	RNU5F-1	ENSG00000202347	RNU1-16P
ENSG00000200169	RNU5D-1	ENSG00000206588	RNU1-28P
ENSG00000275538	RNVU1-19	ENSG00000199568	RNU5A-1
ENSG00000277610	RNVU1-4	ENSG00000200156	RNU5B-1
ENSG00000207349	RNVU1-17	ENSG00000252311	RNU1-103P
ENSG00000207205	RNVU1-15	ENSG00000200903	RNU1-42P
ENSG00000278099	RNVU1-2A	ENSG00000200997	RNU1-85P
ENSG00000277918	RNVU1-28	ENSG00000206687	RNU1-109P
ENSG00000207501	RNVU1-14	ENSG00000238735	RNU7-113P
ENSG00000270722	RNVU1-31	ENSG00000264229	RNU4ATAC
ENSG00000201558	RNVU1-6	ENSG00000201574	RNU1-93P
ENSG00000273768	RNVU1-29	ENSG00000238812	RNU7-127P
ENSG00000274428	RNVU1-25	ENSG00000276027	RNU12
ENSG00000286172	RNVU1-8	ENSG00000207322	RNU1-89P
ENSG00000206585	RNVU1-7	ENSG00000202215	RNU1-51P
ENSG00000199879	RNVU1-22	ENSG00000201910	RNU1-140P
ENSG00000202408	RNVU1-21	ENSG00000206624	RNU1-39P
ENSG00000207340	RNVU1-1	ENSG00000206702	RNU1-11P
ENSG00000252135	RNU1-155P	ENSG00000200597	RNU1-87P
ENSG00000238825	RNVU1-2	ENSG00000206908	RNU1-136P
ENSG00000201183	RNVU1-3	ENSG00000251988	RNU4ATAC18P
ENSG00000274210	RNVU1-27	ENSG00000199846	RNU1-72P
ENSG00000206828	RNVU1-30	ENSG00000207201	RNU1-148P
ENSG00000199672	RNU4-21P	ENSG00000200731	RNU1-124P
ENSG00000223156	RNU2-18P	ENSG00000207110	RNU1-106P
ENSG00000200176	RNU1-19P	ENSG00000251745	RNU7-124P
ENSG00000207175	RNU1-67P		

Table S1. List of snRNA genes used for metaplot analyses, Related to figures 1E, 1F, 2E, 3B, 3D, S1C, S2B, S2D and S2F.
Those highlighted in pink denotes snRNAs with long reads terminating at 3' ends ending in 4<T following DIS3 depletion

TABLE S1

Table S2. primer information

Opening original vector to insert INTS11 homology arms	Sequence
fw	AGTTGCGCAGCCTGAATGGCGA
rv	TACCGAGCTCGAATTCGTAATC
**INTS11 Homology Arms**	GATTACGAATTCGAGCTCGGTAACTCCCTTGGGCCCTGGAGGCCA<br>GGCAGGGTCTCACCGAGCCTCCCCACAGCGTCCTGAAGGACCACT<br>GTGTGCAGCACCTCCCAGACGGCTCTGTGACTGTGGAGTCCGTCC<br>TCCTCCAGGCCGCCGCCCCTTCTGAGGACCCAGGCACCAAGGTGC<br>TGCTGGTCTCCTGGACCTACCAGGTAAGGGGTGACCCCCACCCCA<br>CCGCGGTCAACACAGGTATCAACATTCCTCCCTGTCCTGCACCACC<br>CACAATGCTTTTGCCTCTGCCCTAGGACGAGGAGCTGGGGAGCTT<br>CCTCACATCTCTGCTGAAGAAGGGCCTCCCCCAGGCCCCCAGCTGA<br>GGCCGGCAACTCACCCAGCCGCCACCTCTGCCCTCTCCCAGCTGG<br>ACAGACCCTGGGCCTGCACTTCAGGACTGTGGGTGCCCTGGGTGA<br>ACAGACCCTGCAGGTCCCATCCCTGGGGACAGAGGCCTTGTGTCA<br>CCTGCCTGCCCAGGCAGCTGTTTGCAGCTGAAGAAACAAACTGGTC<br>TCCAGGCTGTCTTGCCTTTATTCCTGGTTAGGGCAGGTGGTCCTA<br>GACAGCAGTTTCCAGTAAAAGCTGAACAAAAGACTACTTGGTACTCT<br>CAGTTGCGCAGCCTGAATGGCGA
**Opening up INTS11 homology vector**	
INTS11-vec fw	CTCTGCCCTCTCCCAGCTGGA
INTS11-vec rv	GCTGGGGGCCTGGGGGAGGC
**INTS11 SMASh amplification**	
INTS11 PCR fw	CCCAGGCCCCCAGCGATGAGATGGAAGAGTGCTCT
INTS11 PCR rv	CTGCAGGATTTCAGGGAGTAGAGAACCTCCCTGTCAGGTA
**INTS11 guide (gRNA) primers**	
INTS11 gRNA fw	CACCGAGAGGGCAGAGGTGGCGGCT
INTS11 gRNA rv	AAACAGCCGCCACCTCTGCCCTCTC
**RNU4-2 Cloning**	
Host plasmid F	AAGGGAATGTGGGAGGTCAG
Host plasmid R	GTCAACGCGTATATCTGGCC
RNU4-2 Insert F	GCATAAGATTCCCCAGCGTC
RNU4-2 Insert R	GGAAACAGCGAAAACTCCGT
Delta T-run F	AAGGGAATGTGGGAGGTCAG
Delta T-run R	CTTTGGATGTATTAATGTGTTAGTTTTAG
RNU4-2 plasmid read-through F	AAGGGAATGTGGGAGGTCAG
RNU4-2 plasmid read-through R	GTTTGCAGCCTCACCTTCTT
**qPCR primers**	
ACTB fw	CATCCGCAAAGACCTGTACG
ACTB rv	CCTGCTTGCTGATCCACATC
INTS1 fw	CCTCATGTACCTGGCCAAGA
INTS1 rv	CATGAGGAGGTTACAGGCCA
RNU4-1 UC fw	CCAATACCCCGCCGTGAC
RNU4-1 UC rv	TGCGAACAAGTACTCTTCAACC
RNU5A-1 UC fw	CTGGTTTCTCTTCAGATCGCA
RNU5A-1 UC rv	CAGAATCTGCTAGTCACTGCT
RNU4-2 500bp fw	ACACTATGTTGGGAACTGGGT
RNU4-2 500bp rv	GGAAACAGCGAAAACTCCGT
RNU4-2 1kb fw	CACTACACCAGCCTCTTCCA
RNU4-2 1kb rv	TTTTCCCAGCACCGTCTTTG
RNU4-2 2kb fw	ACTGCAATCTCCACTTCCCA
RNU4-2 2kb rv	TGAGCCCAGGAGTTTGAGAC
RNU4-2 3kb fw	TATTGGTCAGGCTGGTCTCG
RNU4-2 3kb rv	AACCTTCTCCAGCTGTCCTC
RNU1-1 500bp fw	TCTCTGGGAAGAAAGCAGGG
RNU1-1 500bp rv	ACGGCAGGAGATAGTAGGGA
RNU1-1 1kb fw	GGTTTTGTCCCTGCACTACA
RNU1-1 1kb rv	AGGCTGGTCTTGAACTCCTG
RNU1-1 2kb fw	TCTCTGTTGGGTCGTGTTGA
RNU1-1 2kb rv	GCCACTCTTGCAGATATTGACA
RNU5B-1 300bp fw	CCGGTAATCCCACTGCATTG
RNU5B-1 300bp rv	CATTGTCCATGTGTGCCGAT
RNU5B-1 1.5kb fw	AGAATCGCTTGAACCTGGGA
RNU5B-1 1.5kb rv	CCAGCCTGTGTGATAAAGCC
RNU5D-1 200bp fw	TGTTTGTTGCGAGGTGTGAG
RNU5D-1 200bp rv	GGAAAATCCCTTGAAGCCGG
RNU5D-1 3.5kb fw	TAGCTGAATGTGGTCGTGGT
RNU5D-1 3.5kb rv	TCCTGACCTCATGATCTGCC
RNU1-28P 300bp fw	GTGCTTTCTCCAGGCCAAAG
RNU1-28P 300bp rv	GGACCAGGATTAATTGCCCG
RNU1-28P 2.5kb fw	TTTCACCGTGTCATCCAGGA
RNU1-28P 2.5kb rv	GGGTGACAGCGAGACTTAGT
**siRNAs**	
INTS1	Thermo fisher silencer select # s25211
EXOSC3	Thermo fisher silencer select # s27231
CONTROL	Thermo fisher ON TARGET control #2
**Long-read/EPAP reverse primers**	
Nanopore reverse primer	ACTTGCCTGTCGCTCTATCTTCCCCCCCCCCTTT
EPAP reverse primer (Figure 4H)	ACTTGCCTGTCGCTCTATCTTTTTTTTTTTTTAAA
**RNU5F-1 analysis (supplemental Figure 4G)**	
RT primer	ACTTGCCTGTCGCTCTATCTTTTTTTTTTTTTAAA
RNU5F-1 fw OUT	GGCTGAATGTTCTGTTACTAAAGAG
RNU5F-1 fw IN	ACTAAAGAGAGACGTGTGGGTG
PCR rv	ACTTGCCTGTCGCTCTATCTT

Table S2. Sequences of synthesised DNAs for INTS11 cell line generation, oligonucleotides for qRT-PCR and siRNAs new to this study, Related to STAR Methods

TABLE S2
