# Supplementary material for: Integrator-Dependent and Allosteric/Intrinsic Mechanisms Ensure Efficient Termination of snRNA Transcription
Source: Cell Rep. 2020 Oct 27;33(4):108319. doi: 10.1016/j.celrep.2020.108319 (PMC7610016; doi:10.1016/j.celrep.2020.108319)
Supplement: Document S2. Article plus Supplemental Information [file mmc2.pdf]

Cell Reports

Report

Integrator-Dependent and Allosteric/Intrinsic Mechanisms Ensure Efficient Termination of snRNA Transcription

Graphical Abstract

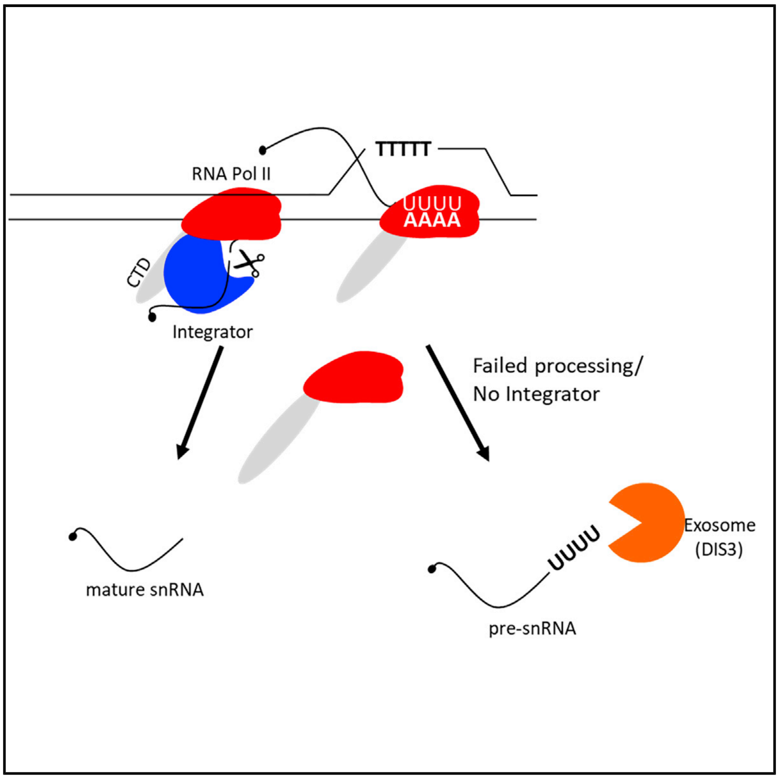

Authors

Lee Davidson, Laura Francis,
Joshua D. Eaton, Steven West

Correspondence

s.west@exeter.ac.uk

In Brief

Davidson et al. show that termination of RNA polymerase II transcription at human snRNA genes can occur by Integrator-dependent and -independent pathways. The latter uses an allosteric/intrinsic mechanism most efficiently detected where T-runs are present in the non-template strand. This is reminiscent of the way many other RNA polymerases terminate.

Highlights

- Integrator-dependent and -independent mechanisms terminate snRNA transcription
- Integrator-independent termination is allosteric/intrinsic in nature
- Sites of allosteric/intrinsic termination often coincide with T-runs
- Some Pol II termination may share features with Pol III/ prokaryotic mechanisms

Davidson et al., 2020, Cell Reports *33*, 108319
October 27, 2020 Crown Copyright © 2020
https://doi.org/10.1016/j.celrep.2020.108319

CellPress

Cell Reports

CellPress OPEN ACCESS

Report

Integrator-Dependent and Allosteric/Intrinsic Mechanisms Ensure Efficient Termination of snRNA Transcription

Lee Davidson,[1] Laura Francis,[1] Joshua D. Eaton,[1] and Steven West[1,2,*]

[1]The Living Systems Institute, University of Exeter, Stocker Rd, Exeter EX4 4QD, UK

[2]Lead Contact

*Correspondence: s.west@exeter.ac.uk

https://doi.org/10.1016/j.celrep.2020.108319

SUMMARY

Many RNA polymerases terminate transcription using allosteric/intrinsic mechanisms, whereby protein alterations or nucleotide sequences promote their release from DNA. RNA polymerase II (Pol II) is somewhat different based on its behavior at protein-coding genes where termination additionally requires endoribonucleolytic cleavage and subsequent 5′→3′ exoribonuclease activity. The Pol-II-transcribed small nuclear RNAs (snRNAs) also undergo endoribonucleolytic cleavage by the Integrator complex, which promotes their transcriptional termination. Here, we confirm the involvement of Integrator but show that Integrator-independent processes can terminate snRNA transcription both in its absence and naturally. This is often associated with exosome degradation of snRNA precursors that long-read sequencing analysis reveals as frequently terminating at T-runs located downstream of some snRNAs. This finding suggests a unifying vulnerability of RNA polymerases to such sequences given their well-known roles in terminating Pol III and bacterial RNA polymerase.

INTRODUCTION

Termination of RNA polymerase II (Pol II) on protein-coding genes requires a polyadenylation signal (PAS) that is bound and endoribonucleolytically processed by a multi-protein cleavage and polyadenylation (CPA) complex with catalytic activity supplied by CPSF73 (Mandel et al., 2006; Proudfoot, 2011, 2016). CPSF73 is crucial because its depletion induces transcriptional read-through that is often hundreds of kilobases in length (Eaton et al., 2020). Its Pol-II-associated cleavage product is degraded 5′→3′ by XRN2, which promotes termination by the so-called "torpedo" model (Eaton et al., 2018; Fong et al., 2015). This mechanism also incorporates allosteric features, defined as modifications to the elongation complex, which slow polymerases down at the end of the gene (Cortazar et al., 2019; Eaton et al., 2020; Eaton and West, 2020). However, Pol II transcribes multiple gene classes where transcriptional termination mechanisms are relatively unexplored. In many of these cases, primary transcripts are endoribonucleolytically cleaved, suggesting some common mechanistic features (Baillat et al., 2005; Fatica et al., 2000).

The spliceosomal small nuclear RNAs (snRNAs) are a prominent Pol-II-transcribed gene class. snRNA precursors are endoribonucleolytically cleaved by the Integrator complex, in which a paralog of CPSF73, called INTS11, provides endoribonuclease activity and promotes transcriptional termination (Baillat et al., 2005; O'Reilly et al., 2014; Skaar et al., 2015). Cleavage is positioned by a 3′ box sequence located after the cut site (Guiro and Murphy, 2017). Recent findings implicate Integrator in promoter-proximal regulation of Pol II transcription (Beckedorff et al., 2020; Elrod et al., 2019; Stadelmayer et al., 2014; Tatomer et al., 2019). Integrator is also involved in the termination of other non-coding transcripts, including enhancer RNAs and promoter upstream transcripts (Beckedorff et al., 2020; Lai et al., 2015; Nojima et al., 2018). Lastly, Integrator mutations are associated with some neurological diseases (Oegema et al., 2017).

In budding yeast, termination of protein-coding gene transcription is similar to that in humans (Kim et al., 2004; West et al., 2004). However, snRNA termination uses the Nrd1-Nab3-Sen1 (NNS) complex, which releases precursors that are then subject to 3′→5′ degradation (Allmang et al., 1999; Schaughency et al., 2014; Steinmetz et al., 2001). Termination of many other RNA polymerases is not associated with prior endo- or exoribonuclease activity and defines 3′ ends directly. For example, Pol III terminates at four or more Ts in the non-template strand, and the prokaryotic RNA polymerase often terminates at similar sequence elements aided by an upstream hairpin sequence (Arimbasseri et al., 2014; Ray-Soni et al., 2016; Zenkin, 2014). The weak thermodynamic stability of rU:dA hybrids may help this process (Martin and Tinoco, 1980). Interestingly, T-runs were identified decades ago as intrinsic terminators of purified human Pol II *in vitro* (Dedrick et al., 1987; Reines et al., 1987).

We have investigated the transcriptional termination mechanism on snRNA genes by asking whether there is obligate coupling to Integrator activities. There is clear involvement of

This is an open access article under the CC BY license (http://creativecommons.org/licenses/by/4.0/).

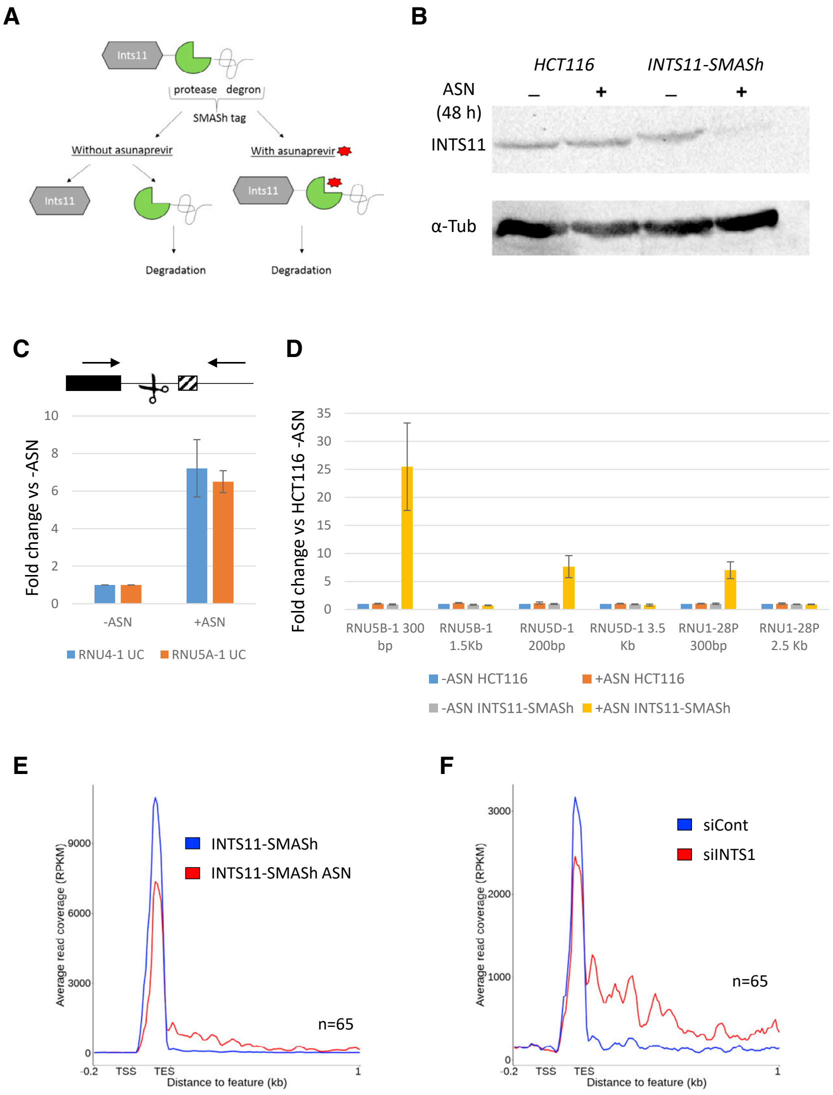

Figure 1. Termination of snRNA Transcription Is Partially Impaired by Integrator Depletion

(A) Schematic of the SMASh tag system. Under normal conditions, the degron-encoded NS3 protease (green) removes the tag and the protein is stable. Addition of ASN (red) prevents this promoting proteosomal degradation.

(B) Western blot demonstrating ASN-dependent depletion of INTS11 after 48 h. Unmodified cells are shown as a control and the lower tubulin blot shows loading.

(C) qRT-PCR analysis of unprocessed (UC, uncleaved) RNU4-1 and RNU5A-1 transcripts in *INTS11-SMASh* cells treated or not with ASN. Levels in treated cells are shown relative to untreated cells after normalizing to spliced actin levels. The schematic shows snRNA (black rectangle), canonical Integrator cleavage site (scissors), downstream 3′ box (hatched box), and primers used (arrows). n = 4, error bars are standard error of the mean (SEM).

(D) qRT-PCR of RNU5 and RNU1 variants in HCT116 or *INTS11-SMASh* cells treated or not with ASN. Primers were used to detect read-through downstream of the annotated 3′ ends of each gene, with the distance indicated under each set of bars. Values are expressed as a fold change versus untreated HCT116 cells after normalizing to spliced actin levels. n = 3, error bars are SEM.

(E) Metagene plot of snRNA genes from nuclear RNA-seq performed on *INTS11-SMASh* cells treated or not with ASN. Signal is reads per kilobase of transcript, per million mapped reads (RPKM). TSS, transcription start site; TES, annotated snRNA 3′ end.

(F) Metagene plot of read-through at all expressed snRNA genes in chromatin-associated RNA-seq from HCT116 cells treated with control or INTS1-specific siRNAs. Signal is RPKM.

Integrator, but termination still occurs without it. In some cases, this involves CPSF73; however, an alternative process seems to directly release 3′ ends for exosome degradation. The characterization of exosome-targeted snRNAs reveals stochastic termination without upstream cleavage and sometimes at T-runs. This feature suggests a common underlying principle for transcriptional termination that, for Pol II, can be applied at snRNA genes.

RESULTS AND DISCUSSION

INTS11 Depletion Delays but Does Not Abolish Termination of snRNA Transcription

To study the role of Integrator in snRNA transcriptional termination, we used CRISPR-Cas9 to Carboxyl (C)-terminally tag endogenous *INTS11* with a small-molecule-assisted shut-off (SMASh) modality (Chung et al., 2015). An internal NS3 protease constitutively cleaves off the SMASh tag but is inhibited by Asunaprevir (ASN) whereupon the tagged factor is degraded (Figure 1A). A constitutively removed tag was selected because the C-terminus of INTS11 is important for functional interactions (Albrecht and Wagner, 2012). HCT116 cells were edited due to their diploid karyotype. PCR confirmed homozygous targeting of *INTS11* with SMASh (Figures S1A and S1B), and western blotting demonstrated depletion of the INTS11 protein after 48 h of ASN treatment (Figure 1B).

We analyzed the impact of INTS11 depletion on snRNA processing by using quantitative reverse transcription and PCR (qRT-PCR) with primers spanning the canonical Integrator cleavage sites on RNU4-1 and RNU5A-1. These species accumulate following ASN treatment, confirming the effectiveness of the system (Figure 1C). Next, we analyzed extended transcripts at three other snRNA genes by using snRNA-proximal and -distal primers, with samples from unmodified HCT116 cells included as a control. ASN treatment increases the levels of snRNA-proximal amplicons in *INTS11-SMASh* cells but not in unmodified cells, showing the specificity of the system (Figure 1D). The

CellPress
OPEN ACCESS

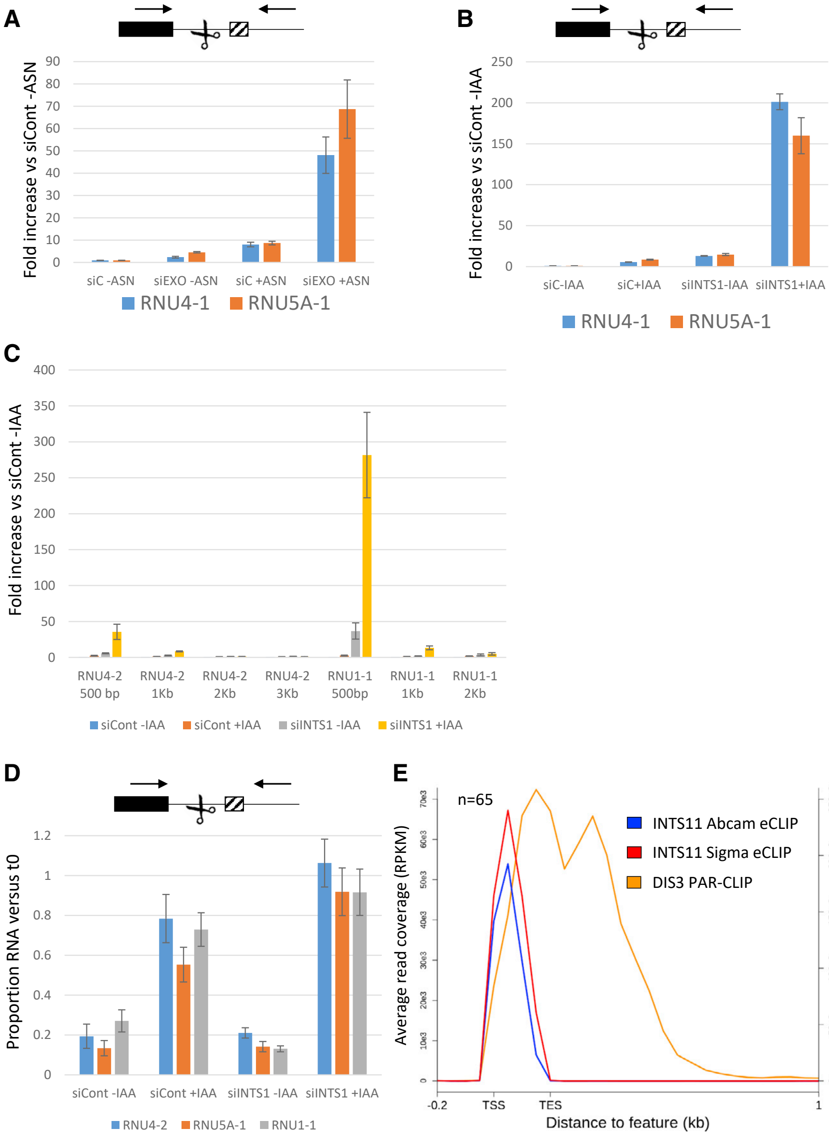

Figure 2. snRNA Precursors Are Targeted for 3′→5′ Degradation by the Exosome

(A) qRT-PCR analysis of *INTS11-SMASh* cells transfected with control or EXOSC3-siRNAs before treatment or not with ASN. Unprocessed RNU4-1 and RNU5A-1 are quantitated relative to control siRNA transfected cells untreated with ASN (siC −ASN) after normalizing to spliced actin. n = 3, error bars are SEM.

(B) qRT-PCR analysis of *DIS3-AID* cells transfected with control or INTS1 siRNAs before treatment or not with auxin (2 h). Unprocessed RNU4-1 and RNU5A-1 are shown relative to control siRNA transfected cells not treated with auxin (siC −IAA) after normalizing to spliced actin. n = 4, error bars are SEM.

(C) qRT-PCR analysis of *DIS3-AID* cells transfected with control or INTS1 siRNAs before treatment or not with auxin (2 h). RNU1-1 and RNU4-2 extended read-through is quantitated relative to siCont-IAA samples after normalizing to spliced actin levels. n = 3, error bars are SEM.

(D) qRT-PCR analysis of snRNA precursor stability in *DIS3-AID* cells transfected with control or INTS1 siRNAs before treatment or not with auxin. Quantitation shows the proportion of precursor remaining after 15 min of actD treatment versus the respective untreated condition (t0). n = 4, error bars are SEM.

(E) Meta-analysis of DIS3 occupancy on snRNA genes as determined by PAR-CLIP performed in HEK293 cells (Szczepińska et al., 2015) and of INTS11 occupancy on snRNAs derived from published eCLIP in HeLa cells (Barra et al., 2020). Data from two different INTS11 antibodies are displayed. The y axes shows RPKM (left for DIS3, right for INTS11).

effect is lower at downstream regions, suggesting that termination occurs in delayed fashion when INTS11 is depleted. Nuclear RNA sequencing (RNA-seq), performed in *INTS11-SMASh* cells, showed this to be generally true for other expressed snRNAs (Figure 1E). An analysis of published Pol II occupancy data confirmed that INTS11 depletion delays but does not abolish snRNA termination (Figure S1C; Stadelmayer et al., 2014).

INTS1 Depletion Delays but Does Not Abolish snRNA Transcriptional Termination

Other Integrator components might be more critical for transcriptional termination than INTS11. To test this, we depleted INTS1 by RNAi—chosen as the largest subunit and separate from the cleavage module (Albrecht et al., 2018). We confirmed INTS1 protein depletion (Figure S1D) and sequenced chromatin-associated RNA from cells treated with control or INTS1 small interfering RNA (siRNAs). Meta-analysis shows that INTS1 depletion increases snRNA read-through but that the effect declines within 1 kb (Figure 1F). An analysis of individual snRNAs/small nucleolar RNAs (snoRNAs) confirms that read-through RNA levels diminish to control levels within a few kb (Figure S1E). This was also the case when *RNU4-2* read-through was assayed following INTS1 and INTS11 co-depletion (Figure S1F). Therefore, although efficient termination of snRNA transcription requires Integrator, other mechanisms can compensate for its depletion.

The Exosome Degrades Precursor snRNAs

Integrator-independent termination should release 3′ ends that might be susceptible to degradation by the 3′→5′ exoribonucleolytic exosome. To investigate this, *INTS11-SMASh* cells were transfected with control siRNAs or siRNAs against the EXOSC3 subunit of the exosome before treatment or not with ASN to degrade INTS11. Unprocessed RNU4-1 and RNU5A-1 precursors accumulate following INTS11 loss as expected (Figure 2A). They also accumulate following EXOSC3 depletion, suggesting that some snRNA precursors are degraded by the exosome. Interestingly, co-depletion of EXOSC3 and INTS11 caused a much larger effect than their individual depletion. A

similar result was seen following RNAi depletion of INTS1 and elimination of the catalytic subunit of the exosome DIS3 by an auxin-inducible degron (AID) tag (Figure 2B). Despite this large accumulation of precursor snRNAs following exosome and Integrator co-depletion, read-through distance remains limited, indicating continued transcriptional termination (Figure 2C). These data are supportive of exosome degradation of snRNA precursors that is more prominent upon reduced Integrator activity.

Another potential explanation for continued termination following Integrator depletion is that residual activity, remaining after knockdown, is sufficient to maintain an Integrator-dependent process. To test the functional extent of INTS1 depletion, *DIS3-AID* cells were transfected with control or INTS1 siRNAs before treatment or not with ASN. Transcription was then inhibited by actinomycin D (actD) for 15 min and unprocessed *RNU4-2*, *RNU5A-1*, and *RNU1-1* transcripts were monitored by qRT-PCR (Figure 2D). In control cells, actD caused a reduction in these precursors, reflecting continued processing/degradation following transcription inhibition. This reduction is less marked following DIS3 loss, which we confirmed using another method of transcriptional inhibition (Figure S2A). As primers span the Integrator cleavage sites, this suggests that DIS3 targets transcripts that escape maturation rather than competing with processing. This idea is supported by recent findings that mature snRNA accumulation is unaffected by exosome depletion (Lardelli and Lykke-Andersen, 2020). Following INTS1 depletion, a substantial fraction of precursors are lost after actD treatment either because of residual Integrator activity or degradation of the resulting unprocessed products. The latter explanation is favored because INTS1 and DIS3 co-depletion prevents most turnover following actD treatment (almost 100% of each precursor remains after 15 min of treatment). This level of functional loss makes it unlikely that residual Integrator fully accounts for termination following its depletion and implicates additional mechanisms.

The generation of precursor snRNA exosome substrates is likely to be common because 3′ flanking transcripts accumulate within just 1 h of DIS3 depletion (Figure S2B). Consistently, an analysis of published PAR-CLIP (photoactivatable ribonucleoside-enhanced crosslinking and immunoprecipitation) shows DIS3 occupancy of snRNAs 3′ flanking RNAs in otherwise unmodified cells (Figure 2E). In contrast, published eCLIP (enhanced CLIP) shows no clear INTS11 crosslinking beyond annotated snRNAs, suggesting that Integrator-independent processes may generate DIS3 substrates (Figure 2E). DIS3 does not occupy transcripts downstream of protein-coding genes, which are unaffected by its loss (Figure S2C; Davidson et al., 2019).

Many Exosome-Targeted snRNA Precursors Are Released by Transcriptional Termination

We hypothesized that the exosome degrades snRNA precursors produced by non-Integrator cleavage activities or by termination itself. CPSF73 could provide alternative endoribonuclease activity and can be rapidly depleted from our previously described *CPSF73-AID* cells by using auxin (Eaton et al., 2020). Although termination of snRNA transcription is unaffected by CPSF73 elimination (Figures S2D and S2E; Eaton et al., 2020), its activity might only be relevant when Integrator is absent. We analyzed chromatin-associated RNA-seq performed in *CPSF73-AID* cells treated with control or INTS1 siRNAs before auxin addition. Co-depletion of CPSF73 with INTS1 enhances read-through at some (e.g., *SNORD13* and *RNU5B-1*) sno/snRNAs (Figure 3A). This finding implies auxiliary cleavage activities may be relevant when Integrator is depleted; however, this effect is not broadly apparent (Figure 3B). An interesting feature of the metagene profiles in Figure 3B is that CPSF73 depletion also reduces the effects of INTS1 loss, indicating some potential cross-talk between these nuclease activities. However, we previously noted reduced nascent transcription of many genes following CPSF73 depletion, with some evidence for this also seen downstream of snRNA genes (Figure S2F; Eaton et al., 2020).

Because CPSF73 activity does not generally explain Integrator-independent termination, we analyzed whether exosome-targeted snRNA precursors are generated by transcriptional termination. We mapped Pol II relative to DIS3 activity by using mammalian native elongating transcript sequencing (mNET-seq), which has single-nucleotide resolution (Nojima et al., 2015), and compared it to DIS3 occupancy uncovered by PAR-CLIP (Szczepińska et al., 2015). Although individual DIS3 and Pol II mNET-seq signals do not necessarily reveal their last possible position, a direct termination mechanism predicts little Pol II occupancy beyond the extremity of the DIS3 PAR-CLIP signal. In contrast, an endoribonuclease-based mechanism classically leads to downstream transcription and 5′→3′ degradation of a 3′ product (Eaton and West, 2020). Individual snRNAs show Pol II occupancy over DIS3-bound regions but much less coverage downstream of them, and a metagene analysis shows a close correlation between the decline of DIS3 and Pol II signals (Figures 3C and 3D). XRN2 elimination has no detectable impact on RNA surrounding these positions, suggesting that there are no substrates for its 5′→3′ exoribonuclease activity (Eaton et al., 2018 and Figure 3E). Although other 5′→3′ exonucleases cannot be completely ruled out, these data suggest that snRNA termination directly releases some DIS3 substrates.

Exosome-Targeted Termination Occurs before snRNA Processing and Sometimes at T-Runs

Allosteric/intrinsic termination in other systems often occurs at T-rich sequences in the non-template strand. To examine the terminal sequence(s) and upstream processing status of snRNA termination products, we used long-read nanopore sequencing on nuclear RNA from *DIS3-AID* cells treated or not with auxin. Long-read protocols normally detect polyadenylated RNA, but snRNA precursors may not have this modification. RNA was, therefore, GI-tailed to provide a common 3′ end to amplify (Figure 4A). To discriminate against RNA from within Pol II (which can also be GI-tailed *in vitro*), reverse -transcription was designed to enrich oligoadenylated 3′ ends, which are well-established features of exosome substrates (LaCava et al., 2005; West et al., 2006; Wyers et al., 2005). RNA-derived products were enriched by GI-tailing, and complete transcripts were recovered from GI-tailed samples, exemplified by GAPDH mRNA (Figure 4B).

Full-length snRNA precursors were also recovered, of which many could be terminated products (Figures 4C–4E). They are

CellPress
OPEN ACCESS

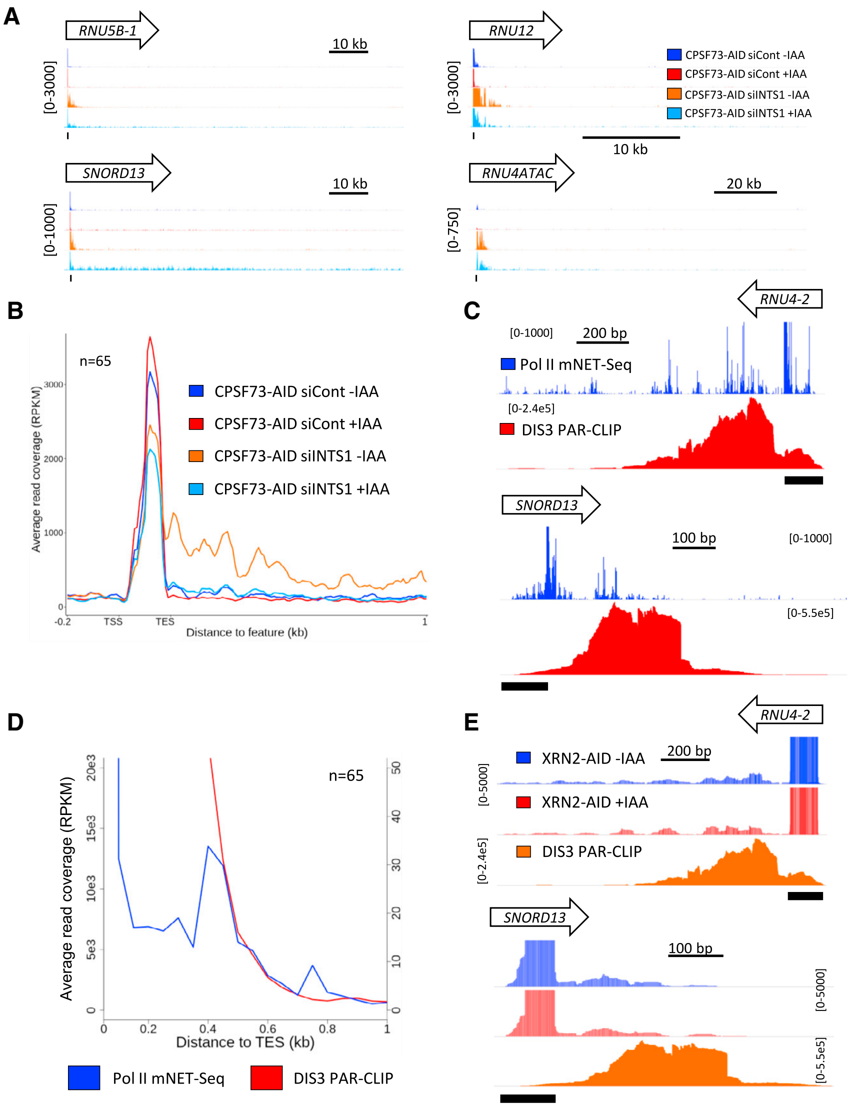

Figure 3. Redundant RNA Cleavage and Direct Termination Generate Some RNA 3′ Ends at snRNA Genes

(A) Integrated Genomics Viewer (IGV) traces of *RNU5B*-1, *SNORD13*, *RNU12*, and *RNU4ATAC* (location indicated by black bar under each trace) from chromatin-associated RNA-seq performed in *CPSF73-AID* cells transfected with control or INTS1 siRNAs before treatment or not with auxin. The y axis shows RPKM.

(B) Metaplot analysis of all snRNAs derived from samples used in (A). The y axis scales are RPKM. The –IAA samples (siCont and siINTS1) are the same as those shown in Figure 1F, as these data were obtained in the same experiment.

(C) IGV tracks of *RNU4-2* and *SNORD13* (location indicated by black bar under each trace) comparing Pol II mNET-seq (blue) and published DIS3 PAR-CLIP (red) signals. mNET-seq signal is mapped to the 3′-most nucleotide of the read deriving from Pol II's active center. "Spikes" of signal represent sites of greater Pol II occupancy. The y axis signals are RPKM.

(D) Metaplot of mNET-seq versus DIS3 PAR-CLIP (Szczepińska et al., 2015) at all expressed snRNAs. The Pol II mNET-seq signal is very high at the final nucleotide of snRNAs because of the detection of their incorporation into spliceosomes (Nojima et al., 2015). Therefore, a zoomed view is provided to highlight nascent signals beyond this region. The y axes show RPKM and signals are in 50-bp bins.

(E) IGV tracks of *RNU4-2* and *SNORD13* (location indicated by black bar under each trace) comparing DIS3 PAR-CLIP signals (Szczepińska et al., 2015) with nuclear RNA-seq obtained after auxin-dependent depletion of XRN2 (1-h depletion) (previously presented in Eaton et al., 2018). The y axis signals are RPKM.

strongly stabilized by DIS3 loss and are not processed by Integrator, which data above anticipated by showing that DIS3 targeted precursors are poorly processed. The 3′ ends show a largely stochastic distribution, arguing against any sequence-defined endoribonucleolytic activity as predicted by our mNET-seq experiment and supporting termination as a possible source. Interestingly, precursors often stack over T-runs in the non-transcribed strand. In many systems, T-runs directly promote (e.g., Pol III) or facilitate (many prokaryotic RNA polymerases) transcriptional termination (Arimbasseri et al., 2014; Ray-Soni et al., 2016). 36% of the snRNAs that we analyzed showed evidence of termination at T > 4, a criteria selected because this is a minimal Pol III terminator (Table S1). Unlike for Pol III, Pol II termination is not inevitable at T-runs, as shown on *RNU5F-1* where reads extend beyond some T-stretches (Figure 4D). This has some analogy with bacterial intrinsic terminators, which culminate in a T-rich sequence but are variably efficient depending on other elements. Some snRNA precursors do not possess extensive T-runs but still exhibit stochastic 3′ termini that are not processed at the upstream canonical Integrator site (Figure S3). 3′ end mapping using an *Escherichia coli* poly(A) polymerase (EPAP) and short-read sequencing gave similar findings to these long-read data (Figures S4A–S4F). Although some 3′ ends detected in these experiments may still represent RNA cleavage sites, the absence of INTS11 cross-linking and the independence of such events from INTS1 argue against Integrator being responsible (Figure 2E andFigure S4G). Moreover, deletion of a single T-run from an *RNU4-2* reporter plasmid caused an enhancement of read-through, further arguing for a function of such elements in termination (Figure 4F).

Here, we identify a transcriptional termination mechanism at snRNAs that does not require canonical Integrator processing. Many such events appear to be allosteric/intrinsic in nature on the basis that 5′→3′ exonuclease activity (XRN2) is not implicated. We suggest that Pol II is generally prone to terminate stochastically downstream of snRNAs, with this activity focused by T-runs. T-runs are intrinsic terminators of other RNA polymerases and of purified Pol II *in vitro* (Dedrick et al., 1987), but we do not rule out the involvement of proteins. In budding yeast,

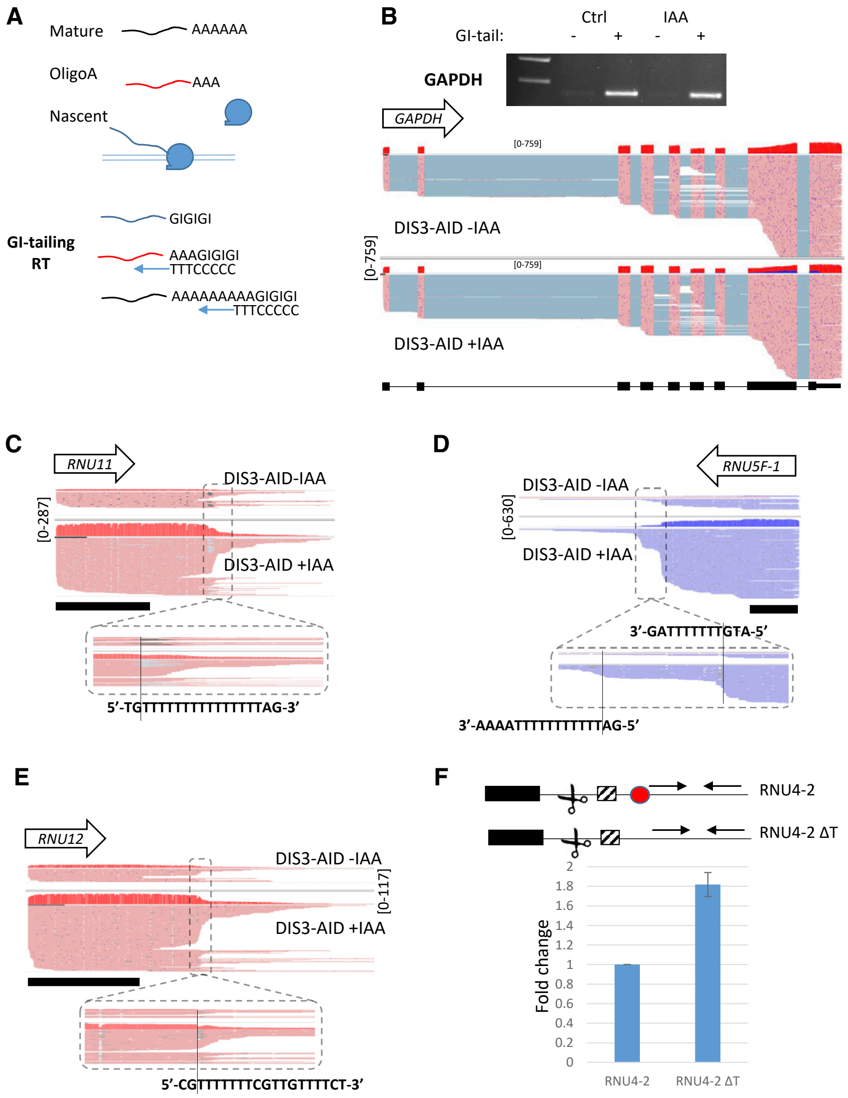

Figure 4. Analysis of Full-Length snRNA Precursors by Long-Read Sequencing

(A) Schematic of long-read sequencing strategy. Nuclei contain mature polyadenylated RNAs with long poly(A) tails (black), terminated oligoadenylated species that might be exosome substrates (red), and unadenylated (blue) transcripts that, for example, might derive from Pol II. GI-tailing allows the sequencing of RNAs with pre-existing poly(A) (black) or oligoA (red) tails following an oligo-dC(T)$_3$-primed cDNA synthesis step.

(B) Agarose gel analysis of GAPDH transcripts isolated from *DIS3-AID* cells treated or not with auxin and/or GI-tailed, as indicated. Also shown is long-read sequencing tracks of GAPDH in GI-tailed samples. Each track shows coverage density at the top (red) scaled as transcripts per million (TPM) with individual reads displayed underneath. Individual reads show exons (pink) linked by introns removed by splicing. White space indicates unmapped regions between reads presumably truncated at their 5′/3′ ends. The blue signal coverage in auxin-treated samples derives from an annotated anti-sense RNA degraded by DIS3.

(C–E) Long-read sequencing tracks of *RNU12*, *RNU11*, and *RNU5F-1* snRNAs from the GI-tailing experiment. Read density (TPM) and individual reads are shown. Regions of focused 3′ termini are indicated with a dashed box, and part of their primary sequence is shown. The black bar under each trace denotes the size and position of each mature snRNA as annotated.

(F) qRT-PCR from HCT116 cells transfected with a plasmid expressing RNU4-2 or a derivative lacking a downstream T-tract (red circle). Primers were used to detect read-through RNA beyond the T-tract, as shown in the schematic. Graph shows fold increase relative to the unmodified construct after normalizing to GFP levels from a co-transfected control plasmid. n = 5. Error bars are SEM.

termination of snRNA transcription uses the Sen1 helicase, and mapping of its terminated transcripts reveals U-rich regions at the 3′ ends that could aid this process (Schaughency et al., 2014). Finally, Integrator is broadly involved in early Pol II termination, including at promoter proximal and enhancer regions (Beckedorff et al., 2020; Lai et al., 2015; Nojima et al., 2018; Stadelmayer et al., 2014). In those cases, its depletion does not prevent termination to the extent that CPSF73 elimination does at protein-coding genes, which might be due to alternative pathways akin to those identified here.

STAR★METHODS

Detailed methods are provided in the online version of this paper and include the following:

- KEY RESOURCES TABLE
- RESOURCE AVAILABILITY
  - Lead contact
  - Materials availability
  - Data and code availability
- EXPERIMENTAL MODEL AND SUBJECT DETAILS
- METHOD DETAILS
  - INTS11-SMASh cell line generation and other cloning
  - Cell culture and experimental manipulation
  - RNA isolation for qRT-PCR
  - Nuclear RNA-seq
  - Chromatin-associated and nucleoplasmic RNA isolation and sequencing
  - GI-tailing and nanopore sequencing
  - EPAP tagging and sequencing
  - mNET-seq
  - ChIP-sequencing
- BIOINFORMATICS
  - Illumina-Sequenced Short-Read Alignment
  - EPAP Read Processing and Alignment

- ○ ONT Basecalling and Read Alignment
- ○ mNET-seq mapping
- ○ Generating Normalized Read Coverage BigWigs
- ○ Metagene Analysis
- ● QUANTIFICATION AND STATISTICAL ANALYSIS

SUPPLEMENTAL INFORMATION

Supplemental Information can be found online at https://doi.org/10.1016/j.celrep.2020.108319.

ACKNOWLEDGMENTS

We thank members of the lab for discussions throughout the project and Pawel Grzechnik for comments on the manuscript. This work was supported by a Wellcome Trust Investigator Award (WT107791/Z/15/Z) and a Lister Institute Research Fellowship held by S.W. We are grateful to The University of Exeter Sequencing Service, where all sequencing was performed; they are supported by a Medical Research Council Clinical Infrastructure Award (MR/ M008924/1), the Wellcome Trust Institutional Strategic Support Fund (WT097835MF), a Wellcome Trust Multi User Equipment Award (WT101650MA), and a Biotechnology and Biological Sciences Research Council Longer and Larger (LoLa) Award (BB/ K003240/1).

AUTHOR CONTRIBUTIONS

L.D. performed the long-read sequencing, made most figures, helped draft the paper, and performed all bioinformatics except for the mNET-seq mapping script, which was provided by J.D.E. L.F. performed experiments relating to INTS11, made the *DIS3-AID* cell line, and performed mNET-seq. S.W, made the *INTS11-SMASh* cell line; performed the EPAP RNA-seq, the chromatin-associated RNA-seq following INTS1 depletion, and the Pol II ChIP-seq in *CPSF73-AID* cells; supervised the project; and wrote the manuscript with input from all authors.

DECLARATION OF INTERESTS

The authors declare no competing interests.

Received: June 2, 2020
Revised: August 25, 2020
Accepted: October 6, 2020
Published: October 27, 2020

REFERENCES

Albrecht, T.R., and Wagner, E.J. (2012). snRNA 3′ end formation requires heterodimeric association of integrator subunits. Mol. Cell. Biol. *32*, 1112–1123.

Albrecht, T.R., Shevtsov, S.P., Wu, Y., Mascibroda, L.G., Peart, N.J., Huang, K.L., Sawyer, I.A., Tong, L., Dundr, M., and Wagner, E.J. (2018). Integrator subunit 4 is a 'Symplekin-like' scaffold that associates with INTS9/11 to form the Integrator cleavage module. Nucleic Acids Res. *46*, 4241–4255.

Allmang, C., Kufel, J., Chanfreau, G., Mitchell, P., Petfalski, E., and Tollervey, D. (1999). Functions of the exosome in rRNA, snoRNA and snRNA synthesis. EMBO J. *18*, 5399–5410.

Arimbasseri, A.G., Rijal, K., and Maraia, R.J. (2014). Comparative overview of RNA polymerase II and III transcription cycles, with focus on RNA polymerase III termination and reinitiation. Transcription *5*, e27639.

Baillat, D., Hakimi, M.A., Näär, A.M., Shilatifard, A., Cooch, N., and Shiekhattar, R. (2005). Integrator, a multiprotein mediator of small nuclear RNA processing, associates with the C-terminal repeat of RNA polymerase II. Cell *123*, 265–276.

Barnett, D.W., Garrison, E.K., Quinlan, A.R., Strömberg, M.P., and Marth, G.T. (2011). BamTools: a C++ API and toolkit for analyzing and managing BAM files. Bioinformatics *27*, 1691–1692.

Barra, J., Gaidosh, G.S., Blumenthal, E., Beckedorff, F., Tayari, M.M., Kirstein, N., Karakach, T.K., Jensen, T.H., Impens, F., Gevaert, K., et al. (2020). Integrator restrains paraspeckles assembly by promoting isoform switching of the lncRNA *NEAT1*. Sci. Adv. *6*, z9072.

Beckedorff, F., Blumenthal, E., daSilva, L.F., Aoi, Y., Cingaram, P.R., Yue, J., Zhang, A., Dokaneheifard, S., Valencia, M.G., Gaidosh, G., et al. (2020). The Human Integrator Complex Facilitates Transcriptional Elongation by Endonucleolytic Cleavage of Nascent Transcripts. Cell Rep. *32*, 107917.

Chung, H.K., Jacobs, C.L., Huo, Y., Yang, J., Krumm, S.A., Plemper, R.K., Tsien, R.Y., and Lin, M.Z. (2015). Tunable and reversible drug control of protein production via a self-excising degron. Nat. Chem. Biol. *11*, 713–720.

Cortazar, M.A., Sheridan, R.M., Erickson, B., Fong, N., Glover-Cutter, K., Brannan, K., and Bentley, D.L. (2019). Control of RNA Pol II Speed by PNUTS-PP1 and Spt5 Dephosphorylation Facilitates Termination by a "Sitting Duck Torpedo" Mechanism. Mol. Cell *76*, 896–908.e894.

Davidson, L., Francis, L., Cordiner, R.A., Eaton, J.D., Estell, C., Macias, S., Caceres, J.F., and West, S. (2019). Rapid Depletion of Dros. Inf. Serv.3, EXOSC10, or XRN2 Reveals the Immediate Impact of Exoribonucleolysis on Nuclear RNA Metabolism and Transcriptional Control. Cell Rep. *26*, 2779–2791.e2775.

Dedrick, R.L., Kane, C.M., and Chamberlin, M.J. (1987). Purified RNA polymerase II recognizes specific termination sites during transcription in vitro. J. Biol. Chem. *262*, 9098–9108.

Eaton, J.D., and West, S. (2020). Termination of Transcription by RNA Polymerase II: BOOM!. Trends Genet. *36*, 664–675.

Eaton, J.D., Davidson, L., Bauer, D.L.V., Natsume, T., Kanemaki, M.T., and West, S. (2018). Xrn2 accelerates termination by RNA polymerase II, which is underpinned by CPSF73 activity. Genes Dev. *32*, 127–139.

Eaton, J.D., Francis, L., Davidson, L., and West, S. (2020). A unified allosteric/torpedo mechanism for transcriptional termination on human protein-coding genes. Genes Dev. *34*, 132–145.

Elrod, N.D., Henriques, T., Huang, K.L., Tatomer, D.C., Wilusz, J.E., Wagner, E.J., and Adelman, K. (2019). The Integrator Complex Attenuates Promoter-Proximal Transcription at Protein-Coding Genes. Mol. Cell *76*, 738–752.e737.

Ewels, P., Magnusson, M., Lundin, S., and Käller, M. (2016). MultiQC: summarize analysis results for multiple tools and samples in a single report. Bioinformatics *32*, 3047–3048.

Fatica, A., Morlando, M., and Bozzoni, I. (2000). Yeast snoRNA accumulation relies on a cleavage-dependent/polyadenylation-independent 3′-processing apparatus. EMBO J. *19*, 6218–6229.

Fong, N., Brannan, K., Erickson, B., Kim, H., Cortazar, M.A., Sheridan, R.M., Nguyen, T., Karp, S., and Bentley, D.L. (2015). Effects of Transcription Elongation Rate and Xrn2 Exonuclease Activity on RNA Polymerase II Termination Suggest Widespread Kinetic Competition. Mol. Cell *60*, 256–267.

Guiro, J., and Murphy, S. (2017). Regulation of expression of human RNA polymerase II-transcribed snRNA genes. Open Biol. *7*, 170073.

Kim, M., Krogan, N.J., Vasiljeva, L., Rando, O.J., Nedea, E., Greenblatt, J.F., and Buratowski, S. (2004). The yeast Rat1 exonuclease promotes transcription termination by RNA polymerase II. Nature *432*, 517–522.

Kim, D., Langmead, B., and Salzberg, S.L. (2015). HISAT: a fast spliced aligner with low memory requirements. Nat. Methods *12*, 357–360.

LaCava, J., Houseley, J., Saveanu, C., Petfalski, E., Thompson, E., Jacquier, A., and Tollervey, D. (2005). RNA degradation by the exosome is promoted by a nuclear polyadenylation complex. Cell *121*, 713–724.

Lai, F., Gardini, A., Zhang, A., and Shiekhattar, R. (2015). Integrator mediates the biogenesis of enhancer RNAs. Nature *525*, 399–403.

Lardelli, R.M., and Lykke-Andersen, J. (2020). Competition between maturation and degradation drives human snRNA 3′ end quality control. Genes Dev. *34*, 989–1001.

Li, H. (2018). Minimap2: pairwise alignment for nucleotide sequences. Bioinformatics *34*, 3094–3100.

Li, H., Handsaker, B., Wysoker, A., Fennell, T., Ruan, J., Homer, N., Marth, G., Abecasis, G., and Durbin, R.; 1000 Genome Project Data Processing Subgroup (2009). The Sequence Alignment/Map format and SAMtools. Bioinformatics *25*, 2078–2079.

Liao, Y., Smyth, G.K., and Shi, W. (2013). The Subread aligner: fast, accurate and scalable read mapping by seed-and-vote. Nucleic Acids Res. *41*, e108.

Liao, Y., Smyth, G.K., and Shi, W. (2014). featureCounts: an efficient general purpose program for assigning sequence reads to genomic features. Bioinformatics *30*, 923–930.

Mandel, C.R., Kaneko, S., Zhang, H., Gebauer, D., Vethantham, V., Manley, J.L., and Tong, L. (2006). Polyadenylation factor CPSF-73 is the pre-mRNA 3′-end-processing endonuclease. Nature *444*, 953–956.

Martin, M. (2011). Cutadapt Removes Adapter Sequences From High-Throughput Sequencing Reads. EMBnet J. *17*, 10–12.

Martin, F.H., and Tinoco, I., Jr. (1980). DNA-RNA hybrid duplexes containing oligo(dA:rU) sequences are exceptionally unstable and may facilitate termination of transcription. Nucleic Acids Res. *8*, 2295–2299.

Nojima, T., Gomes, T., Grosso, A.R.F., Kimura, H., Dye, M.J., Dhir, S., Carmo-Fonseca, M., and Proudfoot, N.J. (2015). Mammalian NET-Seq Reveals Genome-wide Nascent Transcription Coupled to RNA Processing. Cell *161*, 526–540.

Nojima, T., Gomes, T., Carmo-Fonseca, M., and Proudfoot, N.J. (2016). Mammalian NET-seq analysis defines nascent RNA profiles and associated RNA processing genome-wide. Nat. Protoc. *11*, 413–428.

Nojima, T., Tellier, M., Foxwell, J., Ribeiro de Almeida, C., Tan-Wong, S.M., Dhir, S., Dujardin, G., Dhir, A., Murphy, S., and Proudfoot, N.J. (2018). Deregulated Expression of Mammalian lncRNA through Loss of SPT6 Induces R-Loop Formation, Replication Stress, and Cellular Senescence. Mol. Cell *72*, 970–984.e977.

O'Reilly, D., Kuznetsova, O.V., Laitem, C., Zaborowska, J., Dienstbier, M., and Murphy, S. (2014). Human snRNA genes use polyadenylation factors to promote efficient transcription termination. Nucleic Acids Res. *42*, 264–275.

Oegema, R., Baillat, D., Schot, R., van Unen, L.M., Brooks, A., Kia, S.K., Hoogeboom, A.J.M., Xia, Z., Li, W., Cesaroni, M., et al. (2017). Human mutations in integrator complex subunits link transcriptome integrity to brain development. PLoS Genet. *13*, e1006809.

Proudfoot, N.J. (2011). Ending the message: poly(A) signals then and now. Genes Dev. *25*, 1770–1782.

Proudfoot, N.J. (2016). Transcriptional termination in mammals: Stopping the RNA polymerase II juggernaut. Science *352*, aad9926.

Quinlan, A.R., and Hall, I.M. (2010). BEDTools: a flexible suite of utilities for comparing genomic features. Bioinformatics *26*, 841–842.

Ramírez, F., Dündar, F., Diehl, S., Grüning, B.A., and Manke, T. (2014). deepTools: a flexible platform for exploring deep-sequencing data. Nucleic Acids Res. *42*, W187–W191.

Ray-Soni, A., Bellecourt, M.J., and Landick, R. (2016). Mechanisms of Bacterial Transcription Termination: All Good Things Must End. Annu. Rev. Biochem. *85*, 319–347.

Reines, D., Wells, D., Chamberlin, M.J., and Kane, C.M. (1987). Identification of intrinsic termination sites in vitro for RNA polymerase II within eukaryotic gene sequences. J. Mol. Biol. *196*, 299–312.

Robinson, J.T., Thorvaldsdóttir, H., Winckler, W., Guttman, M., Lander, E.S., Getz, G., and Mesirov, J.P. (2011). Integrative genomics viewer. Nat. Biotechnol. *29*, 24–26.

Schaughency, P., Merran, J., and Corden, J.L. (2014). Genome-wide mapping of yeast RNA polymerase II termination. PLoS Genet. *10*, e1004632.

Skaar, J.R., Ferris, A.L., Wu, X., Saraf, A., Khanna, K.K., Florens, L., Washburn, M.P., Hughes, S.H., and Pagano, M. (2015). The Integrator complex controls the termination of transcription at diverse classes of gene targets. Cell Res. *25*, 288–305.

Stadelmayer, B., Micas, G., Gamot, A., Martin, P., Malirat, N., Koval, S., Raffel, R., Sobhian, B., Severac, D., Rialle, S., et al. (2014). Integrator complex regulates NELF-mediated RNA polymerase II pause/release and processivity at coding genes. Nat. Commun. *5*, 5531.

Steinmetz, E.J., Conrad, N.K., Brow, D.A., and Corden, J.L. (2001). RNA-binding protein Nrd1 directs poly(A)-independent 3′-end formation of RNA polymerase II transcripts. Nature *413*, 327–331.

Szczepińska, T., Kalisiak, K., Tomecki, R., Labno, A., Borowski, L.S., Kulinski, T.M., Adamska, D., Kosinska, J., and Dziembowski, A. (2015). DIS3 shapes the RNA polymerase II transcriptome in humans by degrading a variety of unwanted transcripts. Genome Res. *25*, 1622–1633.

Tatomer, D.C., Elrod, N.D., Liang, D., Xiao, M.S., Jiang, J.Z., Jonathan, M., Huang, K.L., Wagner, E.J., Cherry, S., and Wilusz, J.E. (2019). The Integrator complex cleaves nascent mRNAs to attenuate transcription. Genes Dev. *33*, 1525–1538.

West, S., Gromak, N., and Proudfoot, N.J. (2004). Human 5′–> 3′ exonuclease Xrn2 promotes transcription termination at co-transcriptional cleavage sites. Nature *432*, 522–525.

West, S., Gromak, N., Norbury, C.J., and Proudfoot, N.J. (2006). Adenylation and exosome-mediated degradation of cotranscriptionally cleaved pre-messenger RNA in human cells. Mol. Cell *21*, 437–443.

Wyers, F., Rougemaille, M., Badis, G., Rousselle, J.C., Dufour, M.E., Boulay, J., Régnault, B., Devaux, F., Namane, A., Séraphin, B., et al. (2005). Cryptic pol II transcripts are degraded by a nuclear quality control pathway involving a new poly(A) polymerase. Cell *121*, 725–737.

Zenkin, N. (2014). Ancient RNA stems that terminate transcription. RNA Biol. *11*, 295–297.

CellPress
OPEN ACCESS

STAR★METHODS

KEY RESOURCES TABLE

REAGENT or RESOURCE	SOURCE	IDENTIFIER
Antibodies		
INTS1	Bethyl	Cat# A300-361A; RRID: AB_2127258
INTS11	Abbexa	abx005038
Tubulin	Abcam	Cat# Ab7291; RRID: AB_2241126
SPT5	Santa Cruz	Cat# sc-133217; RRID: AB_2196394
RNA pol II (ChIP-seq)	Abcam	Cat# Ab817; RRID: AB_306327
RNA pol II (mNET-seq)	MBL	MABI601 (discontinued)
Chemicals, Peptides, and Recombinant Proteins		
JetPrime	VWR	114-07
Lipofectamine RNAiMAX	Fisher Scientific	13778075
Asunaprevir		
Auxin	Sigma	I2886
Hygromycin	Life Technologies	10687010
Neomycin	Sigma	345810
LUNA qPCR mastermix	NEB	M3003
Protoscript II	NEB	M0368
TURBO DNase	Fisher Scientific	AM2238
Benzonase	Sigma	E1014
Tri-reagent	Fisher Scientific	AM9738
Sheep anti-mouse dynabeads	Thermo fisher	11201D
Doxycycline	Sigma	D3447
Triptolide	Sigma	T3652
Asunaprevir	Cambridge Biosciences	CAY20835-1
Critical Commercial Assays		
RNA-seq library preparation kit	Illumina	20020597
ChIP-seq Sample preparation kit	Cell Signaling Technologies	9003S
ChIP-seq Library preparation kit	NEB	E7103
EPAP experiment RNA-seq library preparation kit	Lexogen	SKU: 016.24
Long read sequencing kit	Oxford Nanopore	SQK-PCS109
Long read sequencing flow cell	Oxford Nanopore	SQK-PCS109
Long read flow cell wash kit	Oxford Nanopore	EXP-WSH002
Poly(A) tail length assay kit (GI tailing)	Thermo Fisher	764551KT
Poly(A) tailing kit (EPAP treatment)	Thermo FIsher	AM1350
Plasmid miniprep kit	QIAGEN	27106
RNA microprep kit	Zymo	R1050
mNET-seq library prep kit	NEB	E7330
Ribozero rRNA depletion kit	Illumina	Discontinued but replaced by 20037135
Deposited Data		
INTS11-SMASh nuclear RNA-seq; DIS3-AID cells long-read sequencing; DIS3-AID cells EPAP RNA-seq; Pol II ChIP-seq in CPSF73-AID cells; mNET-seq analysis of Pol II occupancy	This paper	Gene expression omnibus: GSE150238

(*Continued on next page*)

Continued		
REAGENT or RESOURCE	SOURCE	IDENTIFIER
CPSF73-AID treated with siCont, siINTS1, +/−auxin	This paper and Eaton et al., 2020	Gene expression omnibus: GSE150238; GSE137727
Pol II ChIP-seq control versus INTS11 RNAi	Stadelmayer et al., 2014	Gene expression omnibus: GSE60586
DIS3 PAR-CLIP	Szczepińska et al., 2015	Gene expression omnibus: GSE64332
DIS3-AID cell nuclear RNA-seq ± auxin	Davidson et al., 2019	Gene expression omnibus: GSE120574
XRN2-AID cell nuclear RNA-seq ± auxin	Eaton et al., 2018	Gene expression omnibus: GSE109003
INTS11 eCLIP data	Barra et al., 2020	Gene expression omnibus: GSE148755
Raw western blot/agarose gels	Mendeley Data	
Experimental Models: Cell Lines		
HCT116 *INTS11-SMASh*	This paper	N/A
HCT116 *DIS3-AID*	Davidson et al., 2019	N/A
HCT116 *CPSF73-AID*	Eaton et al., 2020	N/A
HCT116 *XRN2-AID*	Eaton et al., 2018	N/A
Oligonucleotides		
See Table S2	IDT	Table S2, this paper
Recombinant DNA		
pCS6-YFP-SMASh	Addgene	68853
px330	Addgene	42230
P2A-NEO/HYG-SV40 PAS sequences	Eaton et al., 2018	N/A
AID sequence	Davidson et al., 2019	N/A
INTS11-SMASh homology arms	This paper	Table S2
RNU4-2 plasmid	This paper	Table S2
Software and Algorithms		
Bamtools v2.4.0	Barnett et al., 2011	N/A
BEDtools v2.26.0	Quinlan and Hall, 2010	N/A
Cutadapt v1.15;2.9	Martin, 2011	N/A
Deeptools v3.0.2;3.4.2	Ramírez et al., 2014	N/A
FastQC v0.11.9	https://www.bioinformatics.babraham.ac.uk/projects/fastqc/	N/A
featureCounts v2.0.0	Liao et al., 2013, 2014	N/A
Guppy v2.3.7	Oxford Nanopore Technologies LTD	N/A
Hisat2 v.2.1.0	Kim et al., 2015	N/A
IGV v2.8.2	Robinson et al., 2011	N/A
minimap2 v2.16-r934-dirty	Li, 2018	N/A
MultiQC v1.8	Ewels et al., 2016	N/A
Porechop v0.2.4	https://github.com/rrwick/Porechop	N/A
Pychopper v2.0.2	https://github.com/nanoporetech/pychopper	N/A
R v3.6.1	https://cran.r-project.org/	N/A
SAMTools 1.4.1;1.10	Li et al., 2009	N/A
Trim_Galore v0.4.4;0.6.5	http://www.bioinformatics.babraham.ac.uk/projects/trim_galore/	N/A

RESOURCE AVAILABILITY

Lead contact

Further information and requests for resources and reagents should be directed to and will be fulfilled by the Lead Contact, Steven West (s.west@exeter.ac.uk).

Materials availability

New reagents generated in this study are available via the lead contact.

Data and code availability

The accession number for the new sequencing datasets reported in this paper is Gene Expression Omnibus: GSE150238.

EXPERIMENTAL MODEL AND SUBJECT DETAILS

This study employed human HCT116 cell lines (male-derived) and derivatives thereof. These were maintained at 37°C, 5% CO_2 and grown in dulbeccos modified eagle medium (DMEM) containing 10% fetal bovine serum. *INTS11-SMASh* cells were obtained by transfecting a subconfluent 6-well dish with 1ug px330, containing the INTS11 guide RNA sequence, and 1ug each of SMASh neomycin and hygromycin repair constructs using Jetprime. Media was refreshed after 16 hr and after a further 48 hr cells were expanded to a 100mm dish containing 30ug/ml hygromycin and 800ug/ml G418. ~10-14 days later, single colonies were picked and expanded for genomic PCR confirmation of *INTS11* modification. *DIS3-AID* cells are described in detail elsewhere as are the nucleotide sequences of P2A, Neomycin, Hygromycin and the SV40 PAS included in the HDR templates (Davidson et al., 2019; Eaton et al., 2018).

METHOD DETAILS

INTS11-SMASh cell line generation and other cloning

INTS11 homology arms were synthesized by Integrated DNA Technologies and inserted into a pUC-based plasmid using Gibson Assembly. The SMASh tag was PCR isolated from pCS6-YFP-SMASh and inserted into our previously described Hygromycin/Neomycin selection vectors (Eaton et al., 2018), from which an AID tag had been removed by PCR. The resulting insert containing SMASh, a P2A cleavage sequence, the drug marker and an SV40 PAS was isolated by PCR and cloned into the INTS11 homology arm vector, that had been linearized at the stop codon by PCR, using Gibson assembly. The INTS11 guide RNA was designed using Benchling and cloned into px330 digested with BbsI. RNU4-2 sequences were isolated from HCT116 genomic DNA and inserted into a plasmid prepared by PCR amplification of a pcDNA5 FRT/TO plasmid containing the human beta-globin gene. The resulting plasmid replaced beta-globin and the upstream CMV promoter with the RNU4-2 sequence (see Table S2 for primers).

Cell culture and experimental manipulation

For INTS11 depletion ASN was added at 3uM final concentration for 48 hr. For INTS1/EXOSC3 RNAi, 6 well dishes were transfected with control or INTS1 siRNA using Lipofectamine RNAiMAX following the manufacturers protocol. The siRNA transfection was repeated after 24 hr and RNA was isolated 48 hr after that. For DIS3-AID depletion, auxin was added to a final concentration of 500uM. For CPSF73-AID depletion 1ug/ml doxycycline was added for 18 hr before treatment with auxin. ActD and triptolide were used at concentrations of 5ug/ml and 1uM triptolide respectively.

RNA isolation for qRT-PCR

This was generally performed in a 24-well dish using Tri-reagent following the manufacturer guidelines. Isolated RNA was treated with Turbo DNase for 1 hr at 37 degrees before phenol chloroform extraction and ethanol precipitation. 1ug of RNA was reverse transcribed using Protoscript II and 1/50th of the cDNA was used for real-time PCR which was performed on a QIAGEN Rotorgene using LUNA qPCR mastermix.

Nuclear RNA-seq

Nuclei were extracted from a sub-confluent 100mm dish of *INTS11-SMASh* cells after 0 or 48 h of asunaprevir treatment using 4ml HLB (10 mM Tris pH5.5, 10 mM NaCl, 2.5 mM MgCl2, 0.5% NP40) underlayerd with 1ml HLB with 10% sucrose. After spinning for 5 min (500xg) the nuclear pellet was resuspended in 1ml of Tri-reagent and RNA isolated as described for qRT-PCR above. RNA quantity was determined using a Nanodrop 2000 spectrophotometer (Thermo) and its quality was assessed using a Tapestation (Agilent). 1 μg was rRNA-depleted and libraries were generated using TruSeq Stranded Total RNA Library Prep Kit.

Chromatin-associated and nucleoplasmic RNA isolation and sequencing

Nuclei were isolated as above then re-suspended in 100ul NUN1 (20mM Tris-HCl at pH 7.9, 75 mM NaCl, 0.5 mM EDTA, 50% glycerol, 0.85 mM DTT) and incubated for 5 min on ice before the addition of 1 mL of NUN2 buffer (20 mM HEPES at pH 7.6, 1 mM DTT, 7.5 mM MgCl2, 0.2 mM EDTA. 0.3 M NaCl, 1 M urea, 1% NP40). Cells were incubated on ice for 10 mins and shook at 2-3min intervals. After spinning (13000 rpm, 10 mins), nucleoplasmic RNA was phenol chloroform extracted from the supernatant and concentrated by ethanol precipitation. The pellet (containing chromatin-associated RNA) was re-suspended in Trizol and incubated for ~30 mins at 37 degrees. RNA was then isolated as per the manufacturers guidelines. 1ug was prepared and used for RNA sequencing as described for nuclear RNA. Chromatin-associated RNA-seq (Figures 1F, 3A, and 3B) was performed in

CPSF73-AID cells and the control siRNA samples -/+IAA were previously analyzed for protein-coding termination defects (Eaton et al., 2020). INTS1 siRNA samples are first described in the present paper.

GI-tailing and nanopore sequencing

Nuclear RNA extracted from *DIS3-AID* cells treated, or not, with IAA. This was first treated with RiboZero (Illumina) and GI-tailed using the poly(A) tail-length assay kit. The final RNA quantity and average length were determined using an HS Tapestation (Agilent). 50 ng of input RNA was reverse transcribed into cDNA using a modified VNP primer, downstream steps were performed using the cDNA-PCR Sequencing Kit (SQK-PCS109) according to the protocol. For each library, we loaded 100 fmol sequentially on a MK 1 R9 flow cell and sequenced for ~4 h per library on a MinION device. The flow cell was washed between loading and sequencing of each library using a Wash Kit according to the user manual.

EPAP tagging and sequencing

1ug of chromatin-associated or nucleoplasmic RNA was treated or not with EPAP (30 min, 30 degrees) then depleted of rRNA. Sequencing libraries were made using the QUANT-seq REV kit.

mNET-seq

The mNET-seq library protocol was performed as described by Nojima et al. (2016) with the following modifications. Two sub-confluent 150mm dishes of cells were used per sample and chromatin pellets isolated as described above. The chromatin pellet was digested with micrococcal nuclease for 2 mins at 37 degrees. After inactivating the nuclease (25mM EGTA), the supernatant was incubated for 1 hr with magnetic beads (Sheep anti-mouse dynabeads) that had been preincubated for 2 hr with anti-Pol II. Immunoprecipitated RNA was 5′ phosphorylated before purification of fragments between 17-200nt using a Quick-RNA microprep kit (Zymo). Libraries were prepared for sequencing using the NEB Next Small RNA sequencing kit.

ChIP-sequencing

One 100mm *CPSF73-AID* cells were used per condition. ChIP was performed with the Simple ChIP enzymatic chromatin IP kit exactly as described in the product guidelines with one exception. Antibody (5ug Pol II, 8WG16) was coupled to sheep anti-mouse dynabeads instead of the beads included in the kit. Sequencing libraries were generated with the NEBNext® Ultra II DNA Library Prep Kit.

BIOINFORMATICS

Illumina-Sequenced Short-Read Alignment

The quality of demultiplexed raw 50 bp fastq reads was assessed using a combination of FastQC and MultiQC, before adaptor trimming using Trim_Galore (wrapper for Cutadapt) with default settings. Adaptor trimmed reads passing both length and quality cut-offs were then mapped to GRCh38 (Ensembl) using Hisat2 (Kim et al., 2015). Unmapped and multi-mapped and reads with a MAPQ score < 30 were discarded using SAMtools (Li et al., 2009).

EPAP Read Processing and Alignment

Adaptor sequences were first removed from raw 50 bp fastq EPAP reads using Trim_Galore with default setting. Trimmed reads were screened using FastQC and MultiQC to confirm adaptor removal before trimming a second time using Trim_Galore, this time to remove long, non-encoded 3′ poly(A) and poly(T) sequences. Following this, reads were mapped to GRCh38 using Hisat2 (Kim et al., 2015), discarding unmapped, multi-mapped and low MAPRQ scored (< 30) reads using SAMTools (Li et al., 2009).

ONT Basecalling and Read Alignment

The fast5 sequences were base-called and converted to a fastq file format using Guppy before being passed to pychopper to extract full-length reads and orientate them by strand. Adaptor sequences were then removed from full-length reads using porechop and mapped to GRCh38 using minimap2 (Li, 2018), with the following settings:

-a -k 15 -w 5–splice -g 2000 -G 200k -A 1 -B 2 -O 2,32 -E 1,0 -C 9 -z 200 -u f–junc-bonus = 9
–splice-flank = yes–no-long-join–secondary = no

Aligned reads were then filtered to remove unmapped, multi-mapped and low MAPQ (< 30) scored reads using the SAMTools suite (Li et al., 2009).

mNET-seq mapping

The mNET-seq traces used single-nucleotide resolution BAM files corresponding to the 3′ end of the RNA fragment (Nojima et al., 2015).

Generating Normalized Read Coverage BigWigs

For IGV visualization and metagene analysis, libraries were merged with Bamtools (Barnett et al., 2011). Normalized read coverage files of both short-read (Illumina) and long-reads (ONT) were produced using the deepTools at a single nucleotide resolution of sense and antisense separated strands (Ramírez et al., 2014). Illumina reads were normalized to Reads Per Kilobase of transcript, per Million mapped reads (RPKM), whereas ONT reads were normalized to Transcripts-Per-Million (TPM), ignoring reads with a MAPQ score < 30.

Metagene Analysis

For snRNA metagene analysis, expressed snRNA genes (> 5 reads in untreated INTS11 cells) were selected from 157 ensembl annotated snRNA genes present in https://rnacentral.org/ and a window extending 1 kb downstream of each TES was then calculated and the snRNA gene body was scaled to 200bp. To prevent counting of ambiguous reads, snRNA genes near annotated genes (< 1kb downstream of their TES) were discarded using the BEDtools suite (Quinlan and Hall, 2010). Pol III genes were removed. Metagene profiles were then calculated using RPKM normalized read coverage with further graphical processing performed in the R environment.

For CPSF73-AID RNA Pol II ChIP metagene analysis all annotated snRNA genes were selected and a window of 1 kb downstream of the TES was incorporated. Due to differences in signal coverage between the CPSF73-AID samples, the auxin treated profile was scaled to the control profile using the mean signal difference over the gene body (scaled to 200 bp). Further graphical processing of metagene plots was performed in the R environment.

QUANTIFICATION AND STATISTICAL ANALYSIS

qRT-PCR was quantitated using the comparative quantitation function associated with the QIAGEN Rotorgene instrument. Values were first normalized to a loading control (stated in the relevant figure legend) and then samples were compared by quantitating the experimental values relative to the control condition (given the value of 1 by the software). Bars show the average of at least three replicates (exact n provided in figure legends) and error bars show the standard deviation of the mean.

Cell Reports, Volume *33*

Supplemental Information

Integrator-Dependent and Allosteric/Intrinsic Mechanisms Ensure Efficient Termination of snRNA Transcription

Lee Davidson, Laura Francis, Joshua D. Eaton, and Steven West

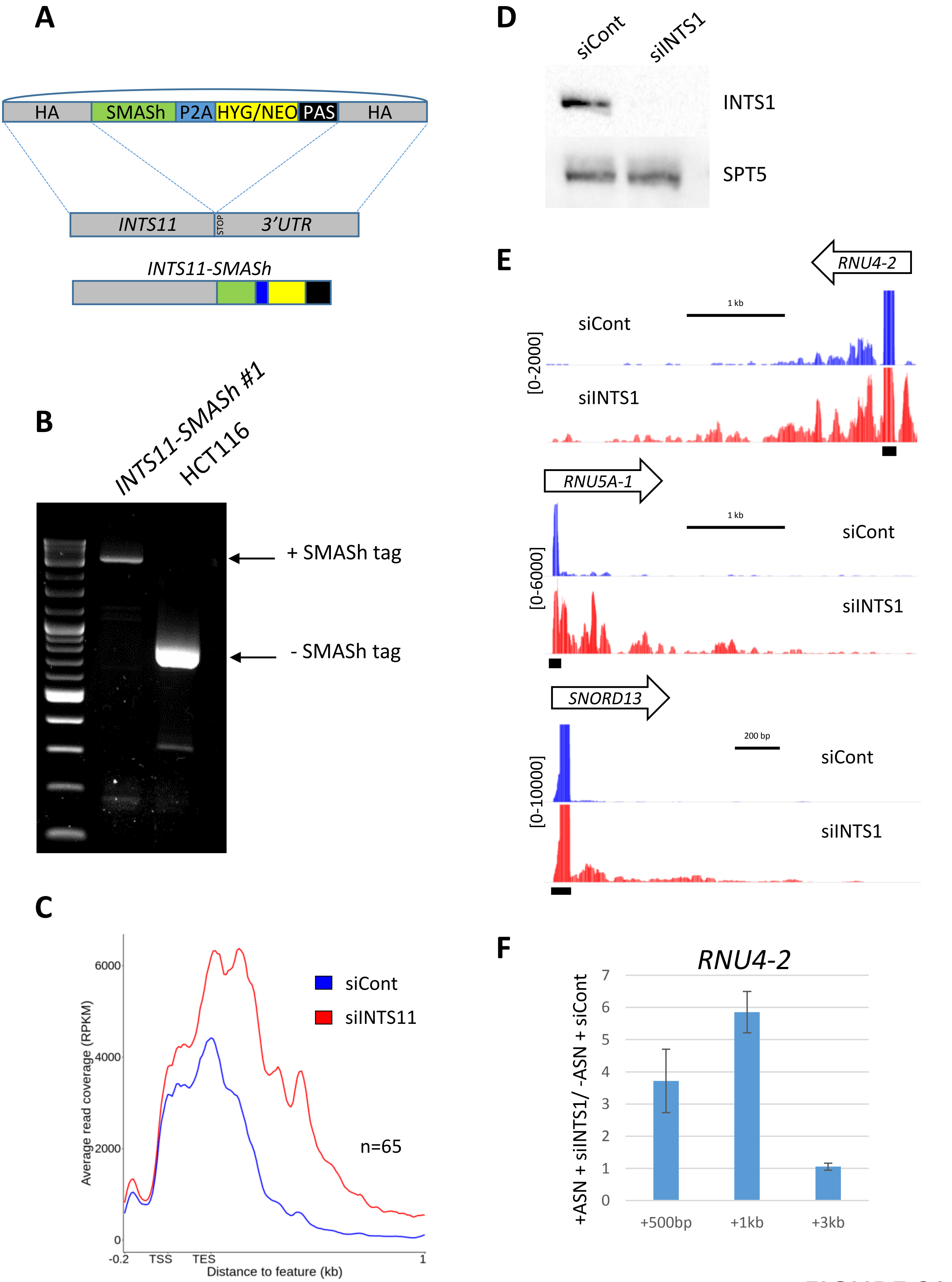
A
HA
SMASh
P2A
HYG/NEO
PAS
HA
INTS11
STOP
3'UTR
INTS11-SMASh
B
INTS11-SMASh #1
HCT116
+ SMASh tag
- SMASh tag
C
Average read coverage (RPKM)
6000
4000
2000
0
-0.2
TSS
TES
1
Distance to feature (kb)
siCont
siINTS11
n=65
D
siCont
siINTS1
INTS1
SPT5
E
RNU4-2
1 kb
siCont
siINTS1
[0-2000]
RNU5A-1
1 kb
siCont
siINTS1
[0-6000]
SNORD13
200 bp
siCont
siINTS1
[0-10000]
F
RNU4-2
+ASN + siINTS1/ -ASN + siCont
7
6
5
4
3
2
1
0
+500bp
+1kb
+3kb

FIGURE S1

FIGURE S1. Integrator depletion delays but does not abolish termination of snRNA transcription, Related to figure 1
A. Schematic of the strategy for inserting the SMASh tag into *INTS11*. The homology-directed repair plasmid is shown with various components coloured and labelled. SMASh is followed by a P2A site, neomycin/hygromycin (Neo/Hyg) resistance markers and an SV40 PAS. While transcribed as a single mRNA, P2A cleavage during translation ensures two separate proteins: INTS11-SMASh and Neo/Hyg.
B. PCR verification of the clone taken forward for study. Primers were used outside of the homology arms such that an upshift in product size would be observed as a result of cassette integration. As can be seen, the modified cell line shows this upshift product with a complete loss of the product indicative of no modification, which is present only in unmodified cells.
C. Metaplot of Pol II ChIP density over-expressed snRNAs in HeLa cells treated with control or INTS11-specific siRNAs. Signal is RPKM. Data are from (Stadelmayer et al., 2014)
D. Western blot of INTS1 levels in *DIS3-AID* cells transfected with control or INTS1-specific siRNAs. SPT5 is also probed for as a loading control.
E. Integrated genome viewer (IGV) snapshots of *RNU4-2, RNU5A-1* and SNORD13 in chromatin-associated RNA-seq obtained after transfection with control or INTS1-specific siRNAs. The signal upstream of *RNU4-2* in the RNAi scenario is read-through from *RNU4-1* positioned shortly upstream. Signal is RPKM. *RNU4-2*, 141bp; *RNU5A-1,*116bp and *SNORD13,* 104bp are shown as a black bar under each trace.
F. qRT-PCR analysis of *INTS11-SMASh* cells transfected with control or INTS1-specific siRNAs before treatment or not with ASN. Levels of RNU4-2 extended read-through was assayed. Position of each amplicon beyond the 3' end processing site is indicated under the graph. Quantitation is relative to non-ASN treated siCont transfected samples after normalising to spliced actin levels. n=3, error bars are SEM.

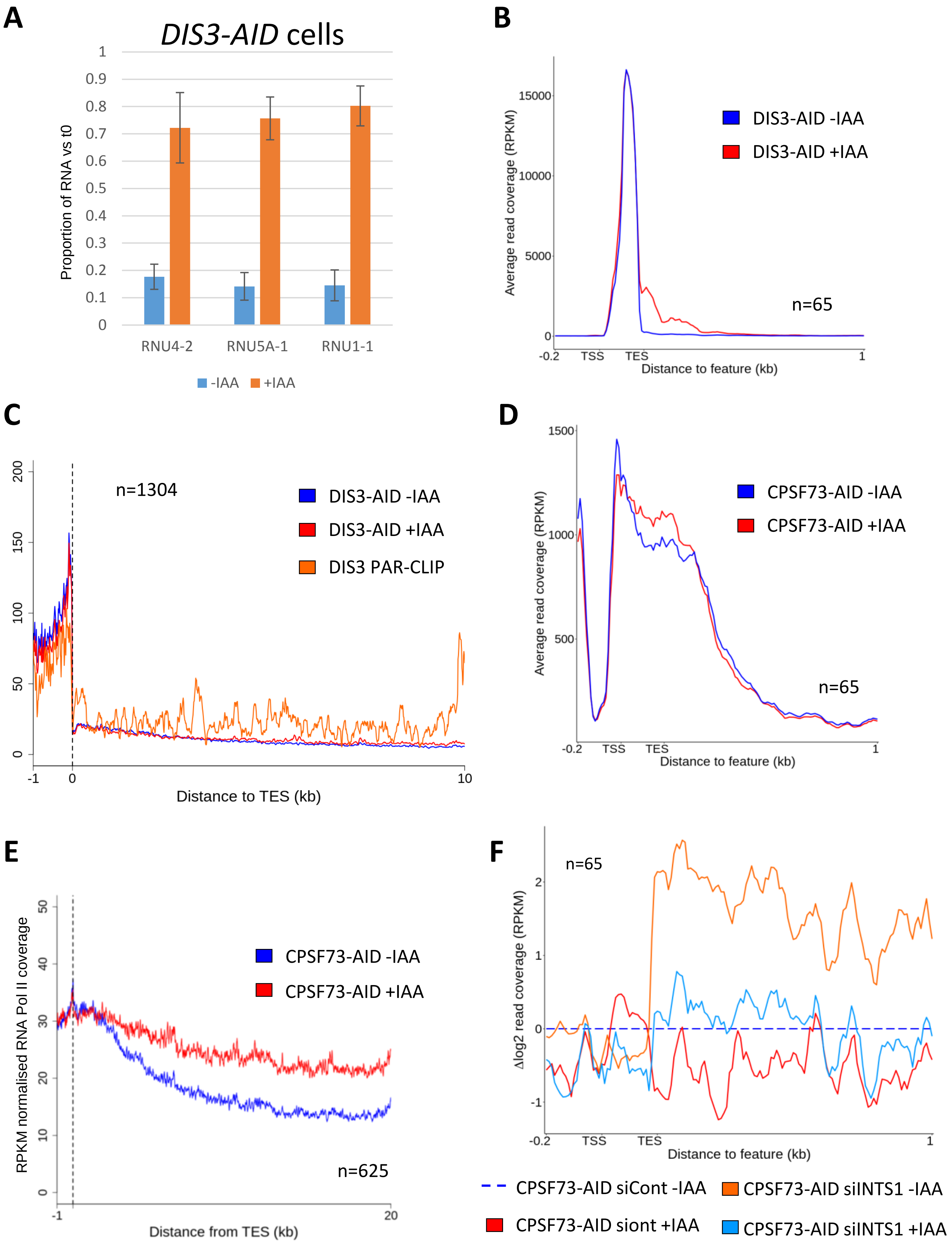
A
DIS3-AID cells
Proportion of RNA vs t0
RNU4-2
RNU5A-1
RNU1-1
-IAA
+IAA
B
Average read coverage (RPKM)
DIS3-AID -IAA
DIS3-AID +IAA
n=65
TSS
TES
Distance to feature (kb)
C
n=1304
DIS3-AID -IAA
DIS3-AID +IAA
DIS3 PAR-CLIP
Distance to TES (kb)
D
Average read coverage (RPKM)
CPSF73-AID -IAA
CPSF73-AID +IAA
n=65
TSS
TES
Distance to feature (kb)
E
RPKM normalised RNA Pol II coverage
CPSF73-AID -IAA
CPSF73-AID +IAA
n=625
Distance from TES (kb)
F
n=65
Δlog2 read coverage (RPKM)
TSS
TES
Distance to feature (kb)
CPSF73-AID siCont -IAA
CPSF73-AID siINTS1 -IAA
CPSF73-AID siont +IAA
CPSF73-AID siINTS1 +IAA

FIGURE S2

FIGURE S2. DIS3 and CPSF73 effects on snRNA processing and read-through RNA, Related to figures 2 and 3

A. qRT-PCR analysis of snRNA precursor stability in *DIS3-AID* cells treated or not with auxin. Quantitation shows the proportion of precursor remaining after 15 mins triptolide treatment versus the respective untreated condition (t0). n=3, error bars are SEM.

B. Meta-analysis of nuclear RNA-seq data obtained in *DIS3-AID* cells treated or not with auxin (1 hr) to assay signal over all expressed snRNA genes (Davidson et al., 2019). Y-axis shows RPKM.

C. Metaplot of all expressed protein-coding genes separated from neighbouring genes by at least 10kb. The region downstream of the PAS (TES) is shown. This shows DIS3-PAR clip signal and reads obtained from our previously published RNA-seq performed in *DIS3-AID* cells treated or not with auxin (1 hr) (Davidson et al., 2019; Szczepinska et al., 2015). Y-axis scale is RPKM. It is clear that DIS3 does not occupy this region and its depletion does not affect read-through RNA levels.

D. Metaplot of snRNA genes generated from the Pol II ChIP-seq performed in *CPSF73-AID* cells treated or not with auxin (3hr). Y-axis scale is RPKM.

E. Metaplot of protein-coding genes that do not contain another gene within 20kb of their TES, generated from the Pol II ChIP-seq performed in *CPSF73-AID* cells treated or not with auxin (3hr). Signal was normalised to gene body levels in each case. Y-axis scale is RPKM. This shows that the negative result in B is not due to incomplete CPSF73 elimination or another experimental failure.

F. Δlog2 representation of the metagene present in main text Figure 3B. This demonstrates the reduced level of 3' flanking RNA in samples depleted of CPSF73 (red line). This likely contributes to the apparent reduced impact of INTS1 depletion in this scenario (light blue trace).

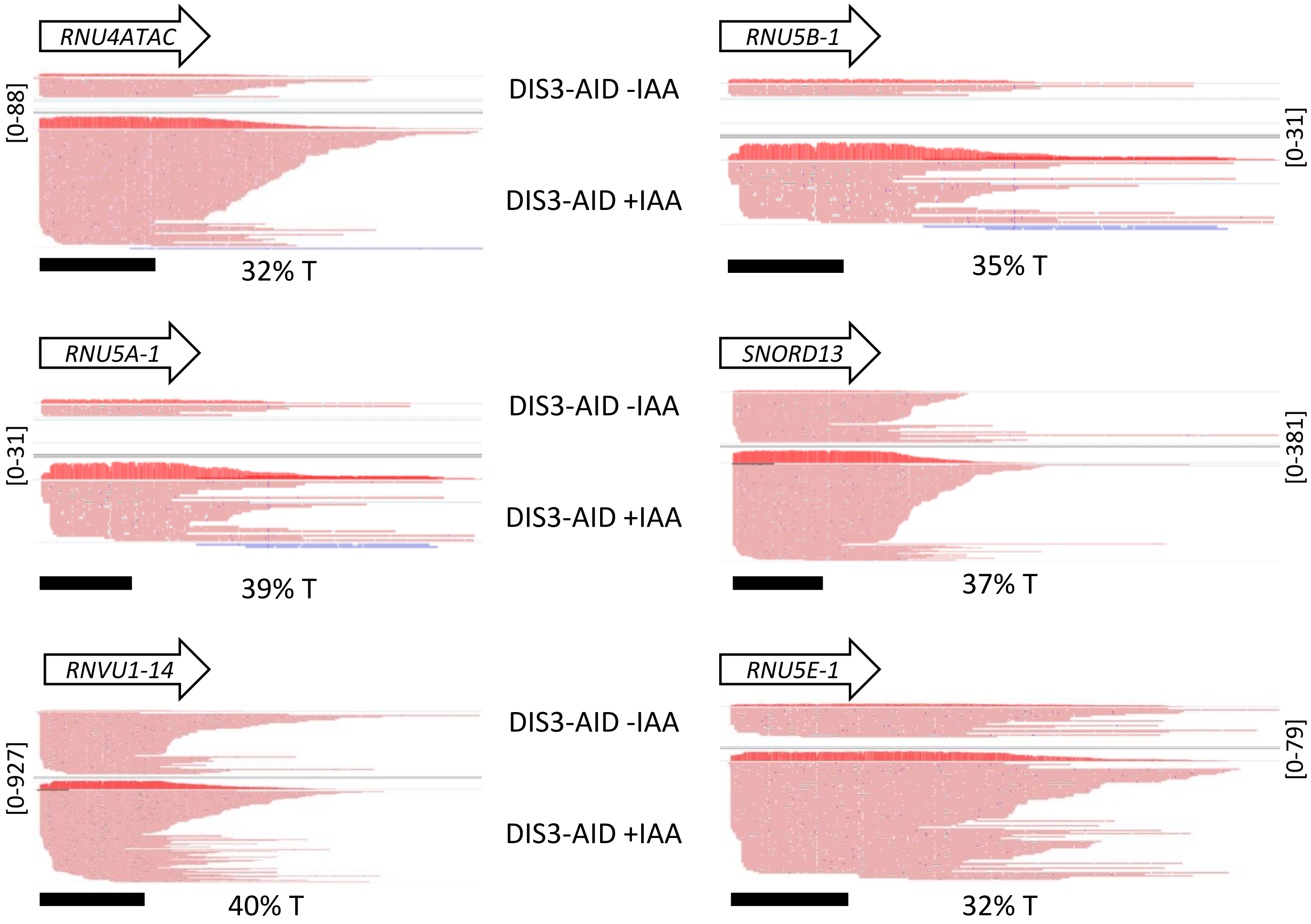

FIGURE S3. Examples of snRNA nanopore sequence traces, Related to figure 4

IGV tracks showing long-read coverage over a collection of sn/snoRNA genes (as labelled) in samples from *DIS3-AID* - cells treated or not with auxin (3hr) to complement those in Figure 4. In all cases, there are stochastic 3' ends that are stabilised by exosome loss. These do not always stack over specific regions though terminator regions have high T content (indicated). By contrast, the 5' ends of most reads map to the expected transcriptional start site regions. Note that some snRNAs show short truncations at their 5' ends. The possibility that these show 5' degradation intermediates cannot be distinguished from the possibility that they represent incomplete cDNA synthesis/5' degradation during RNA isolation. Y-axis scales are TPM. Genes are labelled and their precise position is indicated by the black bars which provide length scale. *RNU4ATAC*, 127bp; *RNU5B-1*, 116bp; *RNU5A-1*, 116bp; *SNORD13*, 104bp; *RNVU1-14*, 164bp; *RNU5E-1*, 120bp.

FIGURE S3

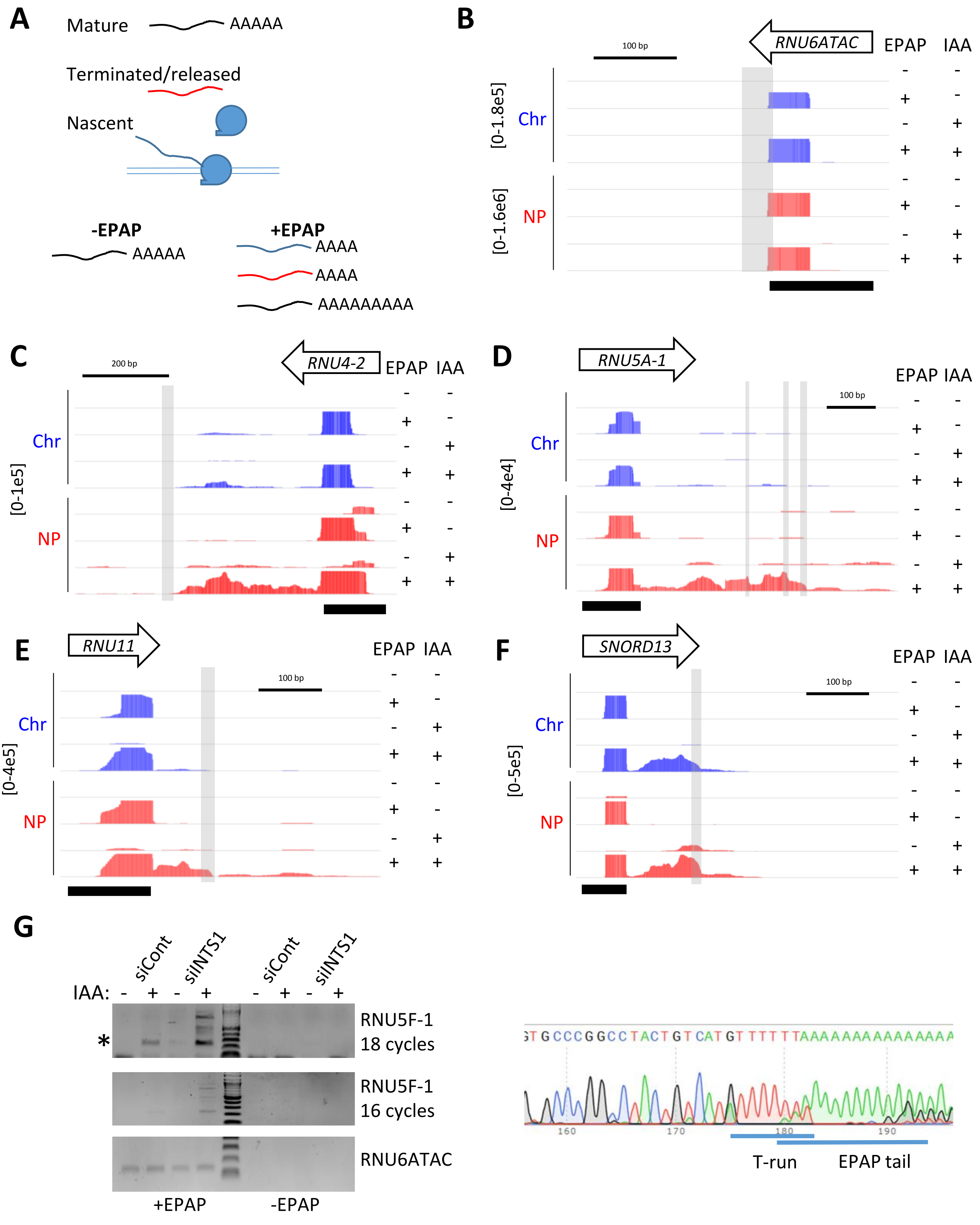

A
Mature
AAAAA
Terminated/released
Nascent
-EPAP
AAAAA
+EPAP
AAAA
AAAA
AAAAAAAAA
B
100 bp
RNU6ATAC
EPAP
IAA
[0-1.8e5]
Chr
[0-1.6e6]
NP
C
200 bp
RNU4-2
EPAP IAA
Chr
[0-1e5]
NP
D
RNU5A-1
100 bp
EPAP IAA
Chr
[0-4e4]
NP
E
RNU11
100 bp
EPAP IAA
Chr
[0-4e5]
NP
F
SNORD13
100 bp
EPAP IAA
Chr
[0-5e5]
NP
G
siCont
siINTS1
siCont
siINTS1
IAA:
*
RNU5F-1
18 cycles
RNU5F-1
16 cycles
RNU6ATAC
+EPAP
-EPAP
GTGCCCGGCCTACTGTCATGTTTTTAAAAAAAAAAAAAA
160
170
180
190
T-run
EPAP tail

FIGURE S4

FIGURE S4. Evidence of termination at T-runs obtained via in vitro polyadenylation and sequencing of 3' ends, Related to figure 4

A. Schematic of an orthogonal 3' end mapping method. Nuclear RNA is treated or not with *E.coli* poly(A) polymerase (EPAP) before 3' end mapping by oligodT primed RNA-seq. Without EPAP, only RNAs with an existing poly(A) tail (black) can be mapped. However, EPAP treatment detects transcripts that are not normally polyadenylated. These might include transcripts isolated from engaged Pol II (blue; chromatin-associated) or other transcripts released from chromatin (red; nucleoplasmic).

B. IGV track for the Pol III-transcribed U6 ATAC gene. Tracks show chromatin (blue) and nucleoplasmic (red) signals deriving from RNAs isolated from *DIS3-AID* cells treated or not with auxin (2hr). EPAP and auxin treatments are indicated beside the traces. U6 ATAC is not normally polyadenylated and is only detected in samples treated with EPAP demonstrating the capacity of this method to differentiate polyadenylated and non-polyadenylated 3' ends. Y-axis scale is RPKM. Gene is labelled and its position on the trace is noted by the black bars which provide length scale. *RNU6ATAC* is 126bp. The shaded region denotes the T-run terminator

C-F. Examples of sn and representative snoRNAs. Labelling is as per B. Transcripts are enriched by EPAP treatment and more abundant when auxin is used demonstrating their normally unadenylated status and susceptibility to DIS3. Signal is more abundant in nucleoplasmic RNA consistent with release of exosome substrates after termination of transcription. However, stabilised reads are also evident in the chromatin-associated fraction consistent with their production by termination (i.e. that they are nascent). We do not know whether there is any subsequent nuclear export/reimport of these species. Note that there is more enrichment of reads further into the 3' flank than for long-read sequencing. This is probably because PCR amplification of longer reads will generally be more biased in favour of shorter cDNAs compared to this short-read approach. Y-axis scale is RPKM. Genes are labelled and their precise position is indicated by the black bars which provide length scale. *RNU4-2*, 141bp; *RNU5A-1*, 115bp; *SNORD13*, 104bp, *RNU12*, 150bp. Shaded areas indicateT-runs.

G. Analysis of 3' terminated precursors from *RNU5F-1* recovered from *DIS3-AID* cells transfected with control or INTS1 siRNAs before treatment or not with auxin. Before PCR amplification, samples were treated or not with EPAP as indicated. Termination products are seen following depletion of DIS3 but are most enriched when DIS3 and INTS1 are co-depleted. The co-depletion reveals additional downstream termination products showing that upstream sites are not sufficient for complete termination. Signals are dependent on EPAP demonstrating that they are genuine 3' ends. Two panels are shown for RNU5F-1 representing different cycle numbers. U6ATAC is a loading control and is detected via the EPAP polyadenylation of its 3' end which is known to be formed at a T-run. The sequencing trace of the asterisked PCR product is shown and confirms its termination at a T-run. Interestingly, the EPAP tail is not evident prior to the 4th T in the tract indicating that a minimum of 4 T's are required to release the RNA at this position.

Ensembl ID	Gene ID	Ensembl ID	Gene ID
ENSG00000199347	RNU5E-1	ENSG00000212413	RNU11-3P
ENSG00000206652	RNU1-1	ENSG00000201616	RNU1-91P
ENSG00000207389	RNU1-4	ENSG00000221439	RNU4ATAC16P
ENSG00000207005	RNU1-2	ENSG00000201435	RNU4-24P
ENSG00000201609	RNU4-28P	ENSG00000202538	RNU4-2
ENSG00000274978	RNU11	ENSG00000200795	RNU4-1
ENSG00000199377	RNU5F-1	ENSG00000202347	RNU1-16P
ENSG00000200169	RNU5D-1	ENSG00000206588	RNU1-28P
ENSG00000275538	RNVU1-19	ENSG00000199568	RNU5A-1
ENSG00000277610	RNVU1-4	ENSG00000200156	RNU5B-1
ENSG00000207349	RNVU1-17	ENSG00000252311	RNU1-103P
ENSG00000207205	RNVU1-15	ENSG00000200903	RNU1-42P
ENSG00000278099	RNVU1-2A	ENSG00000200997	RNU1-85P
ENSG00000277918	RNVU1-28	ENSG00000206687	RNU1-109P
ENSG00000207501	RNVU1-14	ENSG00000238735	RNU7-113P
ENSG00000270722	RNVU1-31	ENSG00000264229	RNU4ATAC
ENSG00000201558	RNVU1-6	ENSG00000201574	RNU1-93P
ENSG00000273768	RNVU1-29	ENSG00000238812	RNU7-127P
ENSG00000274428	RNVU1-25	ENSG00000276027	RNU12
ENSG00000286172	RNVU1-8	ENSG00000207322	RNU1-89P
ENSG00000206585	RNVU1-7	ENSG00000202215	RNU1-51P
ENSG00000199879	RNVU1-22	ENSG00000201910	RNU1-140P
ENSG00000202408	RNVU1-21	ENSG00000206624	RNU1-39P
ENSG00000207340	RNVU1-1	ENSG00000206702	RNU1-11P
ENSG00000252135	RNU1-155P	ENSG00000200597	RNU1-87P
ENSG00000238825	RNVU1-2	ENSG00000206908	RNU1-136P
ENSG00000201183	RNVU1-3	ENSG00000251988	RNU4ATAC18P
ENSG00000274210	RNVU1-27	ENSG00000199846	RNU1-72P
ENSG00000206828	RNVU1-30	ENSG00000207201	RNU1-148P
ENSG00000199672	RNU4-21P	ENSG00000200731	RNU1-124P
ENSG00000223156	RNU2-18P	ENSG00000207110	RNU1-106P
ENSG00000200176	RNU1-19P	ENSG00000251745	RNU7-124P
ENSG00000207175	RNU1-67P		

Table S1. List of snRNA genes used for metaplot analyses, Related to figures 1E, 1F, 2E, 3B, 3D, S1C, S2B, S2D and S2F.
Those highlighted in pink denotes snRNAs with long reads terminating at 3’ ends ending in 4<T following DIS3 depletion

Table S2. primer information

**Opening original vector to insert INTS11 homology arms**	**Sequence**
fw	AGTTGCGCAGCCTGAATGGCGA
rv	TACCGAGCTCGAATTCGTAATC
**INTS11 Homology Arms**	GATTACGAATTCGAGCTCGGTAACTCCCTTGGGCCCTGGAGGCCA GGCAGGGTCTCACCGAGCCTCCCCACAGCGTCCTGAAGGACCACT GTGTGCAGCACCTCCCAGACGGCTCTGTGACTGTGGAGTCCGTCC TCCTCCAGGCCGCCGCCCCTTCTGAGGACCCAGGCACCAAGGTGC TGCTGGTCTCCTGGACCTACCAGGTAAGGGGTGACCCCCACCCCA CCGCGGTCAACACAGGTATCAACATTCCTCCCTGTCCTGCACCACC CACAATGCTTTTGCCTCTGCCCTAGGACGAGGAGCTGGGGAGCTT CCTCACATCTCTGCTGAAGAAGGGCCTCCCCCAGGCCCCCAGCTGA GGCCGGCAACTCACCCAGCCGCCACCTCTGCCCTCTCCCAGCTGG ACAGACCCTGGGCCTGCACTTCAGGACTGTGGGTGCCCTGGGTGA ACAGACCCTGCAGGTCCCATCCCTGGGGACAGAGGCCTTGTGTCA CCTGCCTGCCCAGGCAGCTGTTTGCAGCTGAAGAAACAAACTGGTC TCCAGGCTGTCTTGCCTTTATTCCTGGTTAGGGCAGGTGGTCCTA GACAGCAGTTTCCAGTAAAAGCTGAACAAAAGACTACTTGGTACTCT CAGTTGCGCAGCCTGAATGGCGA
**Opening up INTS11 homology vector**	
INTS11-vec fw	CTCTGCCCTCTCCCAGCTGGA
INTS11-vec rv	GCTGGGGGCCTGGGGGAGGC
**INTS11 SMASh amplification**	
INTS11 PCR fw	CCCAGGCCCCCAGCGATGAGATGGAAGAGTGCTCT
INTS11 PCR rv	CTGCAGGATTTCAGGGAGTAGAGAACCTCCCTGTCAGGTA
**INTS11 guide (gRNA) primers**	
INTS11 gRNA fw	CACCGAGAGGGCAGAGGTGGCGGCT
INTS11 gRNA rv	AAACAGCCGCCACCTCTGCCCTCTC
**RNU4-2 Cloning**	
Host plasmid F	AAGGGAATGTGGGAGGTCAG
Host plasmid R	GTCAACGCGTATATCTGGCC
RNU4-2 Insert F	GCATAAGATTCCCCAGCGTC
RNU4-2 Insert R	GGAAACAGCGAAAACTCCGT
Delta T-run F	AAGGGAATGTGGGAGGTCAG
Delta T-run R	CTTTGGATGTATTAATGTGTTAGTTTTAG
RNU4-2 plasmid read-through F	AAGGGAATGTGGGAGGTCAG
RNU4-2 plasmid read-through R	GTTTGCAGCCTCACCTTCTT
**qPCR primers**	
ACTB fw	CATCCGCAAAGACCTGTACG
ACTB rv	CCTGCTTGCTGATCCACATC
INTS1 fw	CCTCATGTACCTGGCCAAGA
INTS1 rv	CATGAGGAGGTTACAGGCCA
RNU4-1 UC fw	CCAATACCCCGCCGTGAC
RNU4-1 UC rv	TGCGAACAAGTACTCTTCAACC
RNU5A-1 UC fw	CTGGTTTCTCTTCAGATCGCA
RNU5A-1 UC rv	CAGAATCTGCTAGTCACTGCT
RNU4-2 500bp fw	ACACTATGTTGGGAACTGGGT
RNU4-2 500bp rv	GGAAACAGCGAAAACTCCGT
RNU4-2 1kb fw	CACTACACCAGCCTCTTCCA
RNU4-2 1kb rv	TTTTCCCAGCACCGTCTTTG
RNU4-2 2kb fw	ACTGCAATCTCCACTTCCCA
RNU4-2 2kb rv	TGAGCCCAGGAGTTTGAGAC
RNU4-2 3kb fw	TATTGGTCAGGCTGGTCTCG
RNU4-2 3kb rv	AACCTTCTCCAGCTGTCCTC
RNU1-1 500bp fw	TCTCTGGGAAGAAAGCAGGG
RNU1-1 500bp rv	ACGGCAGGAGATAGTAGGGA
RNU1-1 1kb fw	GGTTTTGTCCCTGCACTACA
RNU1-1 1kb rv	AGGCTGGTCTTGAACTCCTG
RNU1-1 2kb fw	TCTCTGTTGGGTCGTGTTGA
RNU1-1 2kb rv	GCCACTCTTGCAGATATTGACA
RNU5B-1 300bp fw	CCGGTAATCCCACTGCATTG
RNU5B-1 300bp rv	CATTGTCCATGTGTGCCGAT
RNU5B-1 1.5kb fw	AGAATCGCTTGAACCTGGGA
RNU5B-1 1.5kb rv	CCAGCCTGTGTGATAAAGCC
RNU5D-1 200bp fw	TGTTTGTTGCGAGGTGTGAG
RNU5D-1 200bp rv	GGAAAATCCCTTGAAGCCGG
RNU5D-1 3.5kb fw	TAGCTGAATGTGGTCGTGGT
RNU5D-1 3.5kb rv	TCCTGACCTCATGATCTGCC
RNU1-28P 300bp fw	GTGCTTTCTCCAGGCCAAAG
RNU1-28P 300bp rv	GGACCAGGATTAATTGCCCG
RNU1-28P 2.5kb fw	TTTCACCGTGTCATCCAGGA
RNU1-28P 2.5kb rv	GGGTGACAGCGAGACTTAGT
**siRNAs**	
INTS1	Thermo fisher silencer select # s25211
EXOSC3	Thermo fisher silencer select # s27231
CONTROL	Thermo fisher ON TARGET control #2
**Long-read/EPAP reverse primers**	
Nanopore reverse primer	ACTTGCCTGTCGCTCTATCTTCCCCCCCCCCTTT
EPAP reverse primer (Figure 4H)	ACTTGCCTGTCGCTCTATCTTTTTTTTTTTTTAAA
**RNU5F-1 analysis (supplemental Figure 4G)**	
RT primer	ACTTGCCTGTCGCTCTATCTTTTTTTTTTTTTAAA
RNU5F-1 fw OUT	GGCTGAATGTTCTGTTACTAAAGAG
RNU5F-1 fw IN	ACTAAAGAGAGACGTGTGGGTG
PCR rv	ACTTGCCTGTCGCTCTATCTT

Table S2. Sequences of synthesised DNAs for INTS11 cell line generation, oligonucleotides for qRT-PCR and siRNAs new to this study, Related to STAR Methods

TABLE S2
